# Mental Health Hospitalizations in Canadian Children, Adolescents, and Young Adults Over the COVID-19 Pandemic

**DOI:** 10.1001/jamanetworkopen.2024.22833

**Published:** 2024-07-08

**Authors:** Nadia Roumeliotis, Matthew Carwana, Ofélie Trudeau, Katia Charland, Kate Zinszer, Mike Benigeri, Mamadou Diop, Jesse Papenburg, Samina Ali, Maryna Yaskina, Gita Wahi, Baudoin Forgeot d’Arc, Sylvana Côté, Manish Sadarangani, Nicole E. Basta, Patricia S. Fontela, Soren Gantt, Terry P. Klassen, Caroline Quach, Quynh Doan

**Affiliations:** 1 Department of Pediatrics, Centre Hospitalier Universitaire Sainte-Justine, Montreal, Quebec, Canada; 2 Faculty of Medicine, University of Montreal, Montreal, Quebec, Canada; 3 Department of Pediatrics, University of British Columbia, Vancouver, British Columbia, Canada; 4 British Columbia Children’s Hospital Research Institute, Vancouver, British Columbia, Canada; 5 Centre Hospitalier Universitaire Sainte-Justine Hospital Research Centre, Montreal, Quebec, Canada; 6 Centre for Public Health Research, University of Montreal, Montreal, Quebec, Canada; 7 School of Public Health, University of Montreal, Montreal, Quebec, Canada; 8 Institut National d’Excellence en Santé et en Services Sociaux, Montreal, Quebec, Canada; 9 Department of Pediatrics, Montreal Children’s Hospital, Montreal, Quebec, Canada; 10 Department of Pediatrics, Faculty of Medicine & Dentistry, University of Alberta, Edmonton, Alberta, Canada; 11 Women and Children’s Health Research Institute, University of Alberta, Edmonton, Alberta, Canada; 12 McMaster Children’s Hospital, Hamilton, Ontario, Canada; 13 McMaster University, Hamilton, Ontario, Canada; 14 Department of Psychiatry, Centre Hospitalier Universitaire Sainte-Justine, Montreal, Quebec, Canada; 15 Department of Epidemiology, Biostatistics and Occupational Health, School of Population and Global Health, Faculty of Medicine and Health Sciences, McGill University, Montreal, Quebec, Canada; 16 Department of Microbiology, Infectious Diseases and Immunology, University of Montreal, Montreal, Quebec, Canada; 17 Children’s Hospital Research Institute of Manitoba, University of Manitoba, Winnipeg, Manitoba, Canada

## Abstract

**Importance:**

The COVID-19 pandemic resulted in multiple socially restrictive public health measures and reported negative mental health impacts in youths. Few studies have evaluated incidence rates by sex, region, and social determinants across an entire population.

**Objective:**

To estimate the incidence of hospitalizations for mental health conditions, stratified by sex, region, and social determinants, in children and adolescents (hereinafter referred to as *youths*) and young adults comparing the prepandemic and pandemic-prevalent periods.

**Design, Setting, and Participants:**

This Canadian population-based repeated ecological cross-sectional study used health administrative data, extending from April 1, 2016, to March 31, 2023. All youths and young adults from 6 to 20 years of age in each of the Canadian provinces and territories were included. Data were provided by the Canadian Institute for Health Information for all provinces except Quebec; the Institut National d’Excellence en Santé et en Services Sociaux provided aggregate data for Quebec.

**Exposures:**

The COVID-19–prevalent period, defined as April 1, 2020, to March 31, 2023.

**Main Outcomes and Measures:**

The main outcome measures were the prepandemic and COVID-19–prevalent incidence rates of hospitalizations for anxiety, mood disorders, eating disorders, schizophrenia or psychosis, personality disorders, substance-related disorders, and self-harm. Secondary measures included hospitalization differences by sex, age group, and deprivation as well as emergency department visits for the same mental health conditions.

**Results:**

Among Canadian youths and young adults during the study period, there were 218 101 hospitalizations for mental health conditions (ages 6 to 11 years: 5.8%, 12 to 17 years: 66.9%, and 18 to 20 years: 27.3%; 66.0% female). The rate of mental health hospitalizations decreased from 51.6 to 47.9 per 10 000 person-years between the prepandemic and COVID-19–prevalent years. However, the pandemic was associated with a rise in hospitalizations for anxiety (incidence rate ratio [IRR], 1.11; 95% CI, 1.08-1.14), personality disorders (IRR, 1.21; 95% CI, 1.16-1.25), suicide and self-harm (IRR, 1.10; 95% CI, 1.07-1.13), and eating disorders (IRR, 1.66; 95% CI, 1.60-1.73) in females and for eating disorders (IRR, 1.47; 95% CI, 1.31-1.67) in males. In both sexes, there was a decrease in hospitalizations for mood disorders (IRR, 0.84; 95% CI, 0.83-0.86), substance-related disorders (IRR, 0.83; 95% CI, 0.81-0.86), and other mental health disorders (IRR, 0.78; 95% CI, 0.76-0.79).

**Conclusions and Relevance:**

This cross-sectional study of Canadian youths and young adults found a rise in anxiety, personality disorders, and suicidality in females and a rise in eating disorders in both sexes in the COVID-19–prevalent period. These results suggest that in future pandemics, policymakers should support youths and young adults who are particularly vulnerable to deterioration in mental health conditions during public health restrictions, including eating disorders, anxiety, and suicidality.

## Key Points

**Question:**

How did hospitalizations for mental health conditions in children, adolescents, and young adults change after the onset of the COVID-19 pandemic?

**Findings:**

In this cross-sectional study of patients aged 6 to 20 years, the overall rate of mental health hospitalizations decreased between the pre–COVID-19 and COVID-19–prevalent periods (from 51.6 to 47.9 per 10 000 person-years). In both sexes, there was a decrease in the hospitalization rate for mood disorders and substance use, despite a rise in hospitalizations for eating disorders, but in females, there was a rise in hospitalizations for anxiety, personality disorders, and suicide and self-harm.

**Meaning:**

Understanding how the pandemic affected children, adolescents, and young adults in Canada is crucial to inform public health policy, and these findings suggest that services geared to females, specifically screening for eating disorders, anxiety, personality disorders, and suicidality, will be important to maintain in future pandemics.

## Teaser

This cross-sectional study assesses changes in hospitalizations for mental health conditions in Canadian children, adolescents, and young adults after the onset of the COVID-19 pandemic.

## Introduction

There is mounting evidence that the health impacts of COVID-19 on children and youths extended beyond direct COVID-19–related illness. While children and youths generally experienced less severe acute medical illness than adults did,^1^ the health of these populations has been indirectly impacted by 2 primary mechanisms: indirect consequences from a caregiver’s impact from COVID-19 and public health measures deployed to minimize hospitalizations and mortality.^2,3^ During the COVID-19 pandemic, over 99% of the world’s children experienced some form of restriction, with 1.5 billion experiencing school closure.^4^ Canada, having the second-highest stringency among the Group of Ten nations, has often been described as having outperformed comparable nations during the pandemic with less adult COVID-19 mortality.^5,6,7^

The pandemic significantly impacted mental health service use of children and youths,^8,9,10^ but there has been inconsistency in how this has been defined and measured. Many studies have evaluated the proportion of emergency department (ED) visits accounting for mental health presentations^9,11^ without accounting for the dramatic drop in overall ED visits in response to public health measures. To our knowledge, research focused on hospitalization rates has not been population based or extended past the initial pandemic wave periods.^11^ Comprehensive studies examining the consequences of the COVID-19 pandemic on youth mental health, inclusive of prepandemic time periods, are limited.

In addition, there are insufficient data to describe important health disparities in service use among youths who are equity deserving, including those with barriers related to access to services secondary to marginalization created by social, environmental, and structural factors. This may be based on income level, race, ethnicity, disability, gender or sex, sexual orientation, or whether youths reside in rural and remote communities. There is concern that groups experiencing structural marginalization may have experienced greater indirect harms, similar to trends seen in direct COVID-19 illness.^12^ While evidence supporting greater social challenges during the pandemic exists, there are few data examining impacts stratified by social position.^13^

The objective of this study was to estimate the difference in crude incidence of hospitalizations for mental health conditions in children and adolescents (hereinafter referred to as *youths*) and young adults, comparing the pre–COVID-19 pandemic and COVID-19–prevalent periods across Canadian regions. Secondary objectives included estimating hospitalization differences by sex, age group, and deprivation, as well as exploring the crude incidence difference in ED visits for these mental health conditions over the study period.

## Methods

### Study Design and Population

This study was a Canadian population-based repeated ecological cross-sectional study, using health administrative data, with the study period from April 1, 2016, to March 31, 2023, and aggregated to the regional level. We evaluated the change in rate of hospitalizations for mental health presentations, comparing the pre–COVID-19 period (April 1, 2016, to March 31, 2020) with the COVID-19–prevalent period (April 1, 2020, to March 31, 2023). These dates accounted for full Canadian fiscal years to be counted in each period. The study population included all school-aged youths and young adults from 6 to 20 years of age in each of the Canadian provinces and territories. The study followed the Reporting of Studies Conducted Using Observational Routinely Collected Health Data (RECORD) statement (an extension of the Strengthening the Reporting of Observational Studies in Epidemiology [STROBE] reporting guideline), provided by the EQUATOR network.^14^ Ethics approval was obtained from the Centre Hospitalier Universitaire Sainte-Justine; consent was not required because of the use of deidentified nationwide data in a study with minimal risk and and in which consent would be impossible to obtain.

### Data Sources

Data were provided by the Canadian Institute for Health Information for 9 provinces and 3 territories and by the Institut National d’Excellence en Santé et en Services Sociaux for Quebec, creating a complete national sample. The Canadian Institute for Health Information provided data from the Discharge Abstract Database (DAD) and the National Ambulatory Care Reporting System (NACRS) database for all provinces except Quebec. The DAD provided complete and comprehensive data on all hospitalizations and diagnoses and coordinated access to demographic, social, and medical administrative data from across multiple Canadian jurisdictions, including pan-Canadian organizations such as Census Canada and Statistics Canada. Exclusions were persons with an invalid or missing medical number including refugees, asylum claimants, persons having left Canada, and visitors. Individual demographic data were linked deterministically to hospitalizations provided by the DAD for all provinces and territories, except Quebec. The Institut National d’Excellence en Santé et en Services Sociaux provided aggregate Quebec data from the Maintenance et Exploitation des Données pour l’Étude de la Clientèle Hospitalière and Banque de Données Commune des Urgences databases. For the incidence rate denominator (events per person-years), estimates of Canada’s population by province, sex, area, and age group were provided by Statistics Canada (estimates on July 1 of each year). Material deprivation quintile estimates were based on the 2016 census. The NACRS database provided demographic and administrative data for the exploratory secondary outcome of ED visits, but data were incomplete. The NACRS ED data are mandated in Quebec, Ontario, Alberta, and Yukon; partially mandated in Prince Edward Island, Nova Scotia, Manitoba, Saskatchewan, and British Columbia; and not mandated in Newfoundland and Labrador, New Brunswick, the Northwest Territories, and Nunavut (eTable 1 in Supplement 1).

### Outcome Measures

The primary outcome measure was hospitalizations for a primary diagnosis of a mental health condition to any inpatient facility (adult or pediatric, including community centers) in Canada. Mental health conditions were identified using a primary diagnosis of the *International Statistical Classification of Diseases and Related Health Problems, Tenth Revision* (*ICD-10*) codes (F10 to F99) for disease-specific categories including anxiety, mood disorders, eating disorders, substance-related use, personality disorders, schizophrenia or psychosis, and other mental health or behavioral disorders. (A full list of *ICD-10* codes is included in eTable 2 in Supplement 1.) For suicide and self-harm, an *ICD-10* code (X60 to X84) in a primary or any secondary position was used to identify hospitalizations across provinces. Transfers between acute care facilities were counted as a single hospital encounter, and the patient’s length of stay was combined. The secondary outcome was ED visits for a primary diagnosis of a mental health condition, with *ICD-10* codes as described above.

### Covariates

Outcomes were aggregated by fiscal year and stratified by region, sex, age, and deprivation. Counts were aggregated by region including Atlantic Canada (Prince Edward Island, Nova Scotia, New Brunswick, and Newfoundland and Labrador); Quebec, Ontario, and Prairies (Manitoba, Saskatchewan, and Alberta); and British Columbia. Sex was defined as sex associated with the provincial medical number. Gender is not collected in Canadian health administrative datasets. Material deprivation was used to evaluate outcome disparities in vulnerable youths and young adults, although these indices are not available in the territories. The material deprivation index included a measure of parent educational level, unemployment, and income. Residence as urban vs rural dwelling was provided, with urban defined as a municipality of 10 000 to 50 000 people at the core. Additional variables included a 2-year look-back period (to April 1, 2014) to identify patients with previous presentations of mental health conditions. This variable was categorized into previous hospitalization, previous ED visit, or no mental health visits in the previous 2 years. Canadian health centers do not collect patient-identified race or ethnicity data.

### Statistical Analysis

The characteristics of hospitalizations were described in frequencies and percentages for both periods, and χ^2^ tests were performed to compare them. To illustrate annual crude incidence by disorder, figures showing rates per 10 000 persons aged 6 to 20 years (population data are in eTable 3 in Supplement 1) stratified by region, sex, and deprivation are presented. A Poisson exact test was used to compare the incidence rate of mental health visits before and during the pandemic for each diagnostic subgroup and sex. Incidence rate ratios (IRRs) with 95% CIs were reported. Data analysis was performed using SAS, version 9.4 (SAS Institute Inc) and R, version 4.3.1^15^ (R Project for Statistical Computing) with a 2-sided significance level of *P* < .05.

## Results

There were 218 101 hospitalizations for mental health during the study period (ages 6 to 11 years: 5.8% [n = 12 742], 12 to 17 years: 66.9% [n = 145 806], and 18 to 20 years: 27.3% [n = 59 553]; 66.0% [n = 143 699] female and 34.0% [n = 74 099] male) among the 6.3 million Canadian youths and young adults. Descriptive statistics of hospitalizations in the pre–COVID-19 and COVID-19–prevalent time periods are presented in the Table and by fiscal year in eTable 4 in Supplement 1. Emergency department visits are presented in eTables 5 and 6 in Supplement 1.

**Table.** Characteristics of Mental Health Hospitalizations Before the COVID-19 Pandemic and During the COVID-19 Pandemic-Prevalent Period

Characteristic	No. (%)			*P* value^c^
	Overall (N = 218 101)	Pre–COVID-19 period (n = 127 273)^a^	COVID-19–prevalent period (n = 90 828)^b^	
Sex^d^				
Female	143 699 (66.0)	81 175 (63.8)	62 524 (69.0)	<.001
Male	74 099 (34.0)	45 988 (36.2)	28 111 (31.0)	
Age group, y				
6-11	12 742 (5.8)	7907 (6.2)	4835 (5.3)	<.001
12-17	145 806 (66.9)	83 720 (65.8)	62 086 (68.4)	
18-20	59 553 (27.3)	35 646 (28.0)	23 907 (26.3)	
Rural address^e^	43 361 (20.6)	25 993 (21.2)	17 368 (19.7)	<.001
Material deprivation index quintile^f^				
1	35 281 (17.6)	19 943 (16.9)	15 338 (18.6)	<.001
2	39 705 (19.8)	23 134 (19.6)	16 571 (20.1)	
3	39 311 (19.6)	22 905 (19.4)	16 406 (19.9)	
4	39 377 (19.6)	23 245 (19.7)	16 132 (19.6)	
5	46 811 (23.3)	28 770 (24.4)	18 041 (21.9)	
Province or territories				
Newfoundland and Labrador	2899 (1.3)	1926 (1.5)	973 (1.1)	<.001
Prince Edward Island	1468 (0.7)	1054 (0.8)	414 (0.5)	
Nova Scotia	3595 (1.6)	2237 (1.8)	1358 (1.5)	
New Brunswick	6422 (2.9)	3791 (3.0)	2631 (2.9)	
Quebec	42 575 (19.5)	24 728 (19.4)	17 847 (19.6)	
Ontario	70 996 (32.6)	40 927 (32.2)	30 069 (33.1)	
Manitoba	7199 (3.3)	4382 (3.4)	2817 (3.1)	
Saskatchewan	14 315 (6.6)	8860 (7.0)	5455 (6.0)	
Alberta	28 928 (13.3)	16 254 (12.8)	12 674 (14.0)	
British Columbia	37 614 (17.2)	21 899 (17.2)	15 715 (17.3)	
Territories^g^	2090 (1.0)	1215 (1.0)	875 (1.0)	
Diagnostic subgroup				
Anxiety	27 780 (12.7)	15 425 (12.1)	12 355 (13.6)	<.001
Eating disorders	11 289 (5.2)	4984 (3.9)	6305 (6.9)	
Mood disorders	51 085 (23.4)	31 007 (24.4)	20 078 (22.1)	
Personality disorders	13 035 (6.0)	6902 (5.4)	6133 (6.8)	
Schizophrenic or psychotic	14 761 (6.8)	8570 (6.7)	6191 (6.8)	
Substance-related disorders	16 593 (7.6)	10 122 (8.0)	6471 (7.1)	
Suicide and self-harm	28 431 (13.0)	15 771 (12.4)	12 660 (13.9)	
Other mental health disorders^h^	55 127 (25.3)	34 492 (27.1)	20 635 (22.7)	
Transfer between centers	30 255 (13.9)	16 264 (12.8)	13 991 (15.4)	<.001
Admitted in pediatric center^i^				
Yes	49 876 (22.9)	27 195 (21.4)	22 681 (25.0)	<.001
No	168 225 (77.1)	100 078 (78.6)	68 147 (75.0)	
Mental health visit in last 2 y				
Emergency department visit	66 319 (30.4)	37 957 (29.8)	28 362 (31.2)	<.001
Hospitalization	54 394 (24.9)	31 899 (25.1)	22 495 (24.8)	
None	97 388 (44.7)	57 417 (45.1)	39 971 (44.0)	

^a^ Pre–COVID-19 period: April 1, 2016, to March 31, 2020.

^b^ COVID-19–prevalent period: April 1, 2020, to March 31, 2023.

^c^ *P* values are from a χ^2^ test of independence between the pre–COVID-19 period and the COVID-19–prevalent period columns for hospitalizations.

^d^ Other sex (n = 303 [0.1%]) is not shown.

^e^ Missing urban or rural designation: n = 7413 (3.4%).

^f^ Missing material deprivation index quintile: n = 17 616 (8.1%); 1, less deprived; 5, most deprived.

^g^ Includes Yukon, Nunavut, and Northwest Territories because of low counts for hospitalization data from the Canadian Institute for Health Information.

^h^ Includes adjustment disorders, dissociative disorders, attention-deficit/hyperactivity disorder, tic disorders, and other behavioral disorders (see eTable 2 in Supplement 1).

^i^ Defined as a tertiary center with a pediatric intensive care unit.

### Hospitalizations

Of the 218 101 hospitalizations, the relative proportion of hospitalizations for mental health disorders increased in youths aged 12 to 17 years from 65.8% (83 720 of 127 273 hospitalizations) in the 4 years before the pandemic to 68.4% (62 086 of 90 828 hospitalizations) in the 3-year COVID-19–prevalent period, while mental health hospitalizations for age groups 6 to 11 years (from 6.2% [n = 7097] to 5.3% [n = 4835]) and 18 to 20 years (from 28.0% [n = 35 646] to 26.3% [n = 23 907]) decreased in the same time periods (*P* < .001) (Table). Females accounted for 63.8% (n = 81 175) of mental health hospitalizations during the 4-year prepandemic period, and the proportion increased to 69.0% (n = 62 524) during the 3-year COVID-19–prevalent period (*P* < .001) (Table). A greater proportion of hospitalizations were in pediatric centers in the COVID-19–prevalent period (25.0% [n = 22 681]) vs before the pandemic (21.4% [n = 27 195]), accounting for a greater proportion of transfers (COVID-19–prevalent period: 15.4% [n = 13 991] vs before the pandemic: 12.8% [n = 16 264]; *P* < .001). During the prepandemic 4 years, mental health hospitalizations of patients in the most materially deprived quintile accounted for a larger proportion of hospitalizations (24.4% [n = 28 770]) compared with the least materially deprived quintile (16.9% [n = 19 943]), and this difference was reduced in COVID-19–prevalent years (most materially deprived quintile: 21.9% [n = 18 041] vs least materially deprived quintile: 18.6% [n = 15 338]; *P* < .001), possibly due to a higher number of hospitalizations in the least materially deprived group (Table).

**Figure 1.**
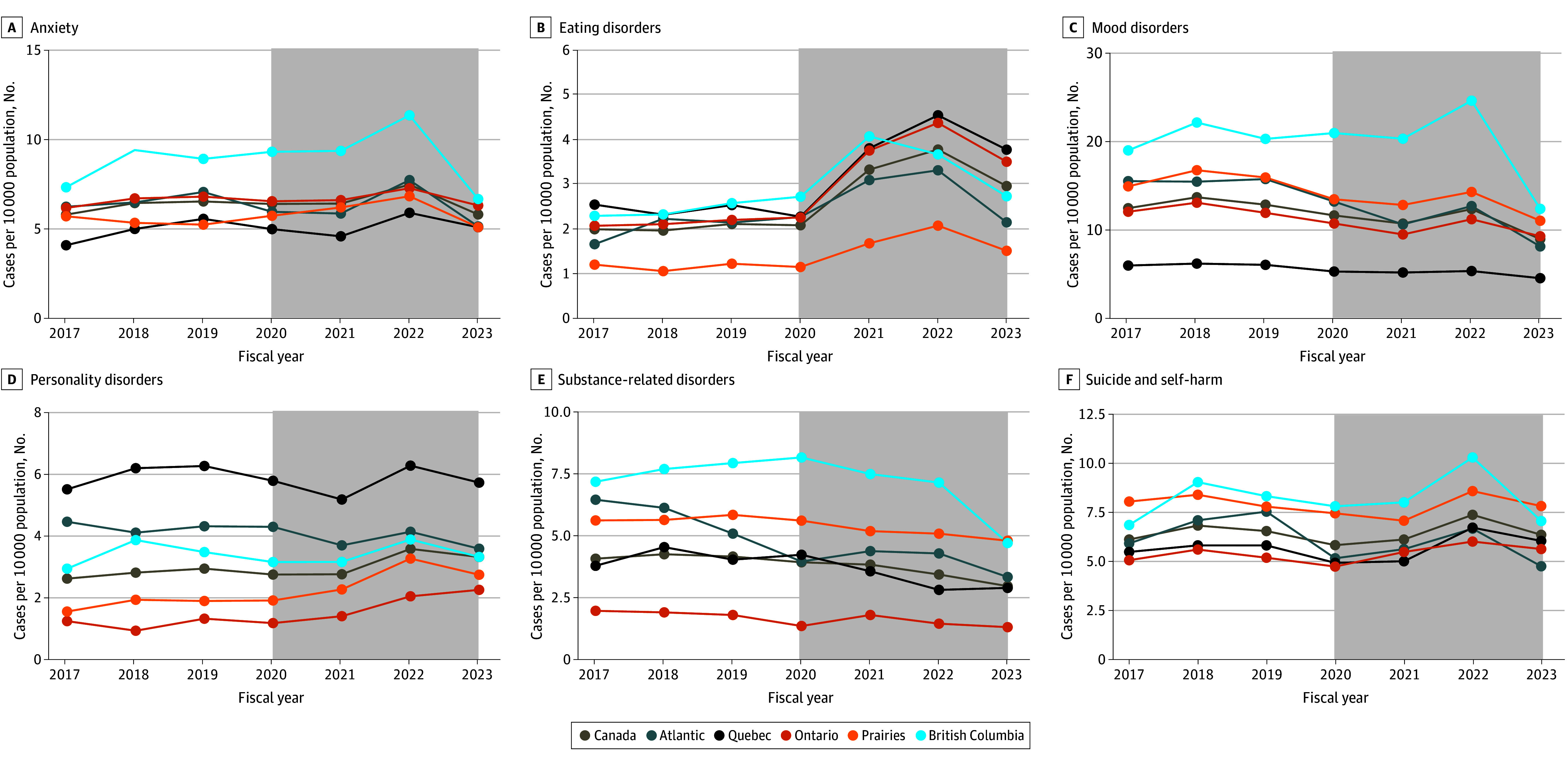
Rates of Mental Health Hospitalizations by 10 000 Person-Years for Each Diagnostic Subgroup, Stratified by Region Gray shaded areas indicate the COVID-19–prevalent period from April 1, 2020, to March 31, 2023. Points correspond to aggregate data by fiscal year and are displayed at the end of the fiscal year (March 31). Territories are not included in the figure because of scale and low counts. (Visits in the territories accounted for 1% of mental health visits in the prepandemic period and during the pandemic.) Atlantic indicates Prince Edward Island, Nova Scotia, New Brunswick, and Newfoundland and Labrador; Prairies, Manitoba, Saskatchewan, and Alberta.

Overall, the rate of mental health hospitalizations decreased by 7.2% (IRR, 0.93; 95% CI, 0.92-0.94) between pre–COVID-19 and COVID-19–prevalent years (from 51.6 to 47.9 per 10 000 person-years). Changes in the annual crude incidence of hospitalizations per 10 000 person-years, by region (excluding the territories) and by mental health disorder, are presented in Figure 1 (with territories and schizophrenic or psychotic and other mental health disorders in eFigure 1 in Supplement 1). Regional patterns were similar between pre–COVID-19 and COVID-19–prevalent periods, with an increase in eating disorders and a decrease in mood (IRR, 0.84; 95% CI, 0.83-0.86), substance-related use (IRR, 0.83; 95% CI, 0.79-0.88), and other mental health (IRR, 0.78; 95% CI, 0.75-0.80) disorders in all regions (Figure 1). Some regional increases in anxiety, mood disorders, and suicide and self-harm for British Columbia were found in 2022, which returned to pre–COVID-19 levels in 2023.

**Figure 2.**
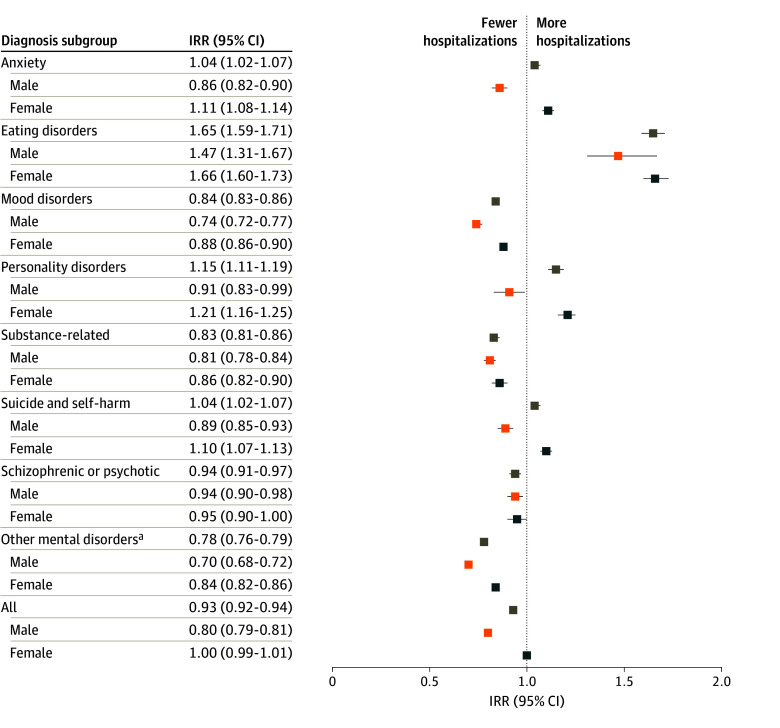
Forest Plot of Incidence Rate Ratios (IRRs) of COVID-19–Prevalent vs Pre–COVID-19 Hospitalization Rates for Mental Health Disorders, Stratified by Sex ^a^Includes adjustment disorders, dissociative disorders, attention-deficit/hyperactivity disorder, tic disorders, and other behavioral disorders (eTable 2 in Supplement 1).

**Figure 3.**
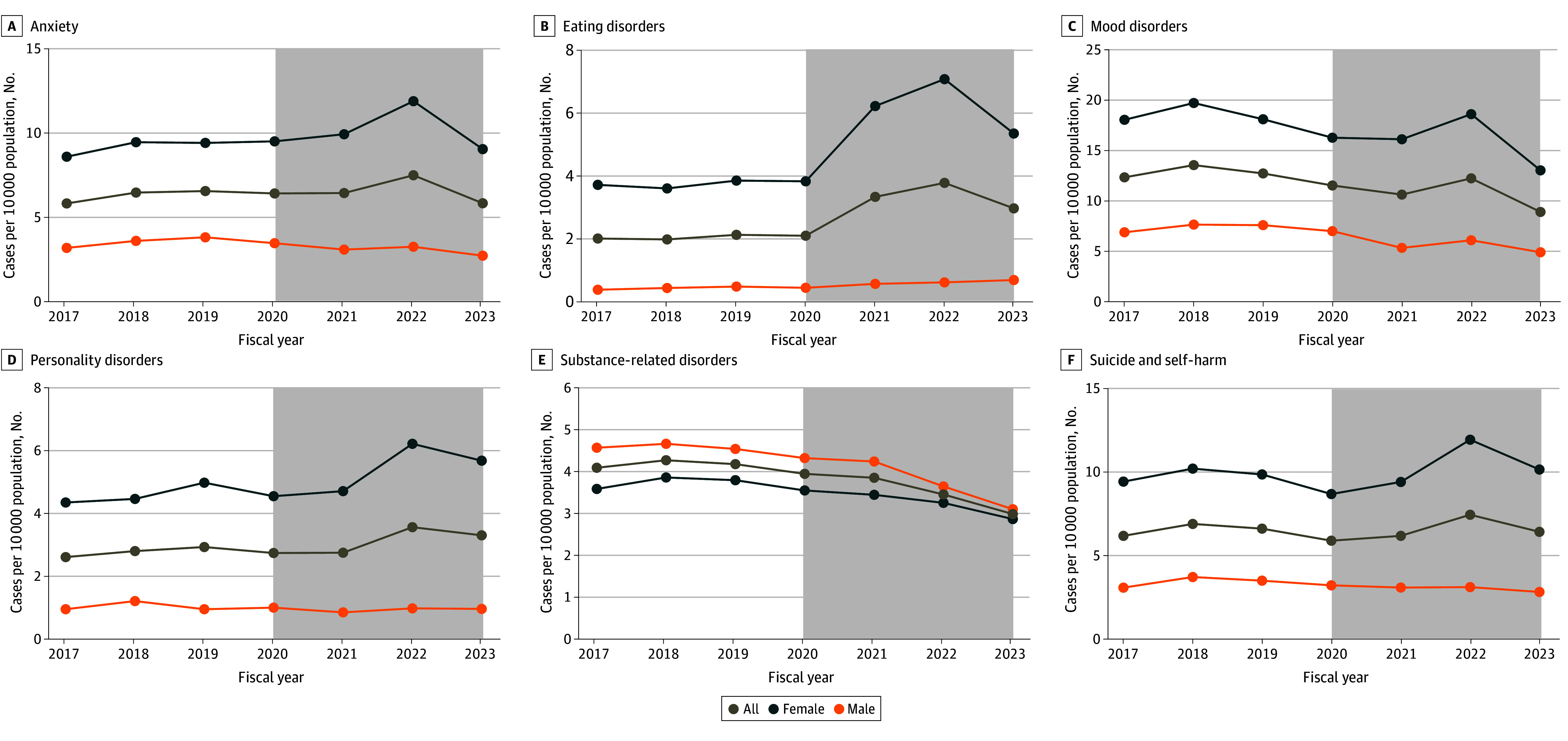
Rates of Mental Health Hospitalizations by 10 000 Person-Years for Each Diagnostic Subgroup, Stratified by Sex Gray shaded areas indicate the COVID-19–prevalent period from April 1, 2020, to March 31, 2023. Points correspond to aggregate data by fiscal year and are displayed at the end of the fiscal year (March 31).

When stratifying by sex and age (Figure 2), there were significant increases among females in anxiety (IRR, 1.11; 95% CI, 1.08-1.14), eating disorders (IRR, 1.66; 95% CI, 1.60-1.73), suicide and self-harm (IRR, 1.10; 95% CI, 1.07-1.13), and personality disorders (IRR, 1.21; 95% CI, 1.16-1.25) in the COVID-19–prevalent period, with a peak incidence in the 2021 to 2022 fiscal year (Figure 3). These increases occurred in disorders that were already more common among females, and the increase was attributable primarily to that seen in those 12 to 17 years of age (eFigure 2 in Supplement 1). Among males, hospitalizations for eating disorders also increased (IRR, 1.47; 95% CI, 1.31-1.67). In the COVID-19–prevalent period, there was a decreasing incidence in mental health disorders more common in males, such as substance use (Figure 3). In the 12- to 17-year-old age group, there were also relative increases in personality disorders but relative decreases in mood, substance use, and other disorders (eFigure 2 in Supplement 1). In young adults 18 to 20 years of age, there were transient increases in schizophrenic or psychotic disorders and personality disorders, which are more common in this age category, but overall decreases in substance use and other disorders (eFigure 2 in Supplement 1). There were no major changes in the hospitalizations for mental health in the 6- to 12-year-old age group (eFigure 2 in Supplement 1).

**Figure 4.**
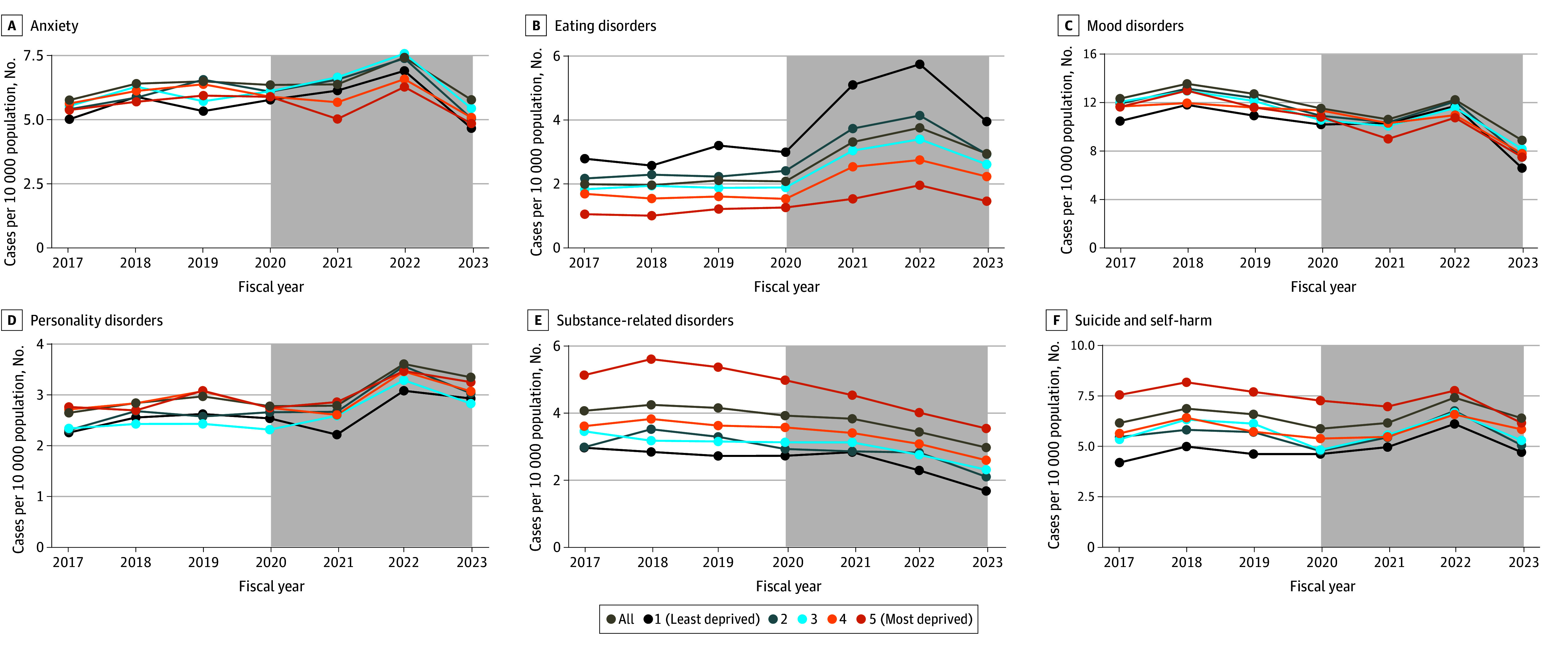
Rates of Mental Health Hospitalizations by 10 000 Person-Years for Each Diagnostic Subgroup, Stratified by Material Deprivation Quintile Gray shaded areas indicate the COVID-19–prevalent period from April 1, 2020, to March 31, 2023. Points correspond to aggregate data by fiscal year and are displayed at the end of the fiscal year (March 31).

Living in a rural community was associated with a higher incidence rate of mental health hospitalizations for all disorders except eating disorders (eFigure 3 in Supplement 1) in both the pre–COVID-19 and COVID-19–prevalent periods. During both periods, youths and young adults in the most materially deprived quintile had a higher incidence of substance-related disorders, schizophrenic or psychotic disorders, suicide and self-harm, and other disorders, although the difference in the material deprivation quintiles narrowed in the COVID-19–prevalent period (Figure 4). For eating disorders, the highest increase in COVID-19–prevalent hospitalizations occurred in the least materially deprived quintile, whereas the lowest increase occurred in the most materially deprived quintile (Figure 4).

Incidence rate ratios of COVID-19–prevalent vs pre–COVID-19 hospitalization by diagnosis stratified by sex are presented in Figure 2. Among females during the pandemic, a statistically significant increase in the rate of hospitalizations was found: 11.2% for anxiety (IRR, 1.11; 95% CI, 1.08-1.14), 20.7% for personality disorders (IRR, 1.21; 95% CI, 1.16-1.25), 10.0% for suicide and self-harm (IRR, 1.10; 95% CI, 1.07-1.13), and 66.2% for eating disorders (IRR, 1.66; 95% CI, 1.60-1.73) (Figure 2). In males, there was a statistically significant 47.5% increase in eating disorders during the pandemic (IRR, 1.47; 95% CI, 1.31-1.67), as well as a 64.6% increase for all hospitalizations for eating disorders (IRR, 1.65; 95% CI, 1.59-1.71) (Figure 2). In both sexes, there was a statistically significant decrease (ranging from 6.0% to 22.2%) during the pandemic-prevalent period in mood disorders (IRR, 0.84; 95% CI, 0.81-0.86), substance-related disorders (IRR, 0.83; 95% CI, 0.81-0.86), schizophrenic or psychotic disorders (IRR, 0.94; 95% CI, 0.91-0.97), and other mental health disorders (IRR 0.78; 95% CI, 0.76-0.79) (Figure 2).

### Emergency Department Visits

Of the available data, there were 881 765 ED visits for mental health during the study period. Similar to hospitalizations, females accounted for a greater proportion of available ED visits during the 3-year COVID-19–prevalent period (65.8% [n = 232 147]) compared with the 4-year pre–COVID-19 period (60.0% [n = 316 953]) (*P* < .001), along with youths aged 12 to 17 years (COVID-19–prevalent period: 52.7% [n = 186 363] vs pre–COVID-19 period: 46.0% [n = 243 272]; *P* < .001) (eTables 5 and 6 in Supplement 1). While the incidence of ED visits by disorder could not be calculated due to provinces with absent or incomplete data, patterns in ED data for reporting centers, except Yukon, revealed an increase in ED visits for eating disorders during the COVID-19–prevalent period (eFigure 4 in Supplement 1) and a decrease in mood disorders, psychosis, and substance-related disorders in provinces with complete ED data. In provinces with complete data, COVID-19–prevalent vs pre–COVID-19 ED visit IRRs rose for eating disorders in both sexes (IRR, 2.13; 95% CI, 2.03-2.23), along with a rise in suicide and self-harm in females (IRR, 1.10; 95% CI, 1.08-1.12) and other disorders in females (IRR, 1.08; 95% 1.07-1.10) (eTable 7 and eFigure 4 in Supplement 1). Apart from eating disorders, male ED visits for each diagnostic category showed a significant decrease (ranging from 12.1% to 45.6%) during the COVID-19–prevalent period compared with the prepandemic period (eg, suicide and self-harm: from 29.0 to 25.5 visits per 10 000 person-years; IRR, 0.88 [95% CI, 0.86-0.90] and substance-related use: from 45.3 to 24.7 per 10 000 person-years; IRR, 0.54 [95% CI, 0.53-0.55]) (eTable 7 in Supplement 1). For both sexes, there were statistically significant decreases in ED visits for mood disorders (IRR, 0.82; 95% CI, 0.81-0.83), personality disorders (IRR, 0.86; 95% CI, 0.84-0.88), and substance-related disorders (from 47.0 to 26.1 visits per 10 000 person-years; IRR, 0.56 [95% CI, 0.55-0.56]) (eTable 7 in Supplement 1).

## Discussion

In this cross-sectional study, we found that the hospitalization rate for eating disorders increased by two-thirds (64.6%) in the COVID-19–prevalent time period compared with before the COVID-19 pandemic, along with increases in personality disorders, anxiety, and suicide and self-harm across provinces. This pattern was largely attributable to the changes seen in females, predominantly those aged 12 to 17 years, who accounted for the majority of the admissions. While the changes in these mental health presentations are concerning, we also found that the COVID-19–prevalent period was associated with decreased rates of hospitalizations (and ED visits) in both sexes for mood disorders, substance use, and other mental health disorders.

We know that mental health-related ED visits, particularly in females, were already on the rise in Canada prior to the COVID-19 pandemic.^16^ This may have been due to an increased prevalence of disease or to increased awareness and literacy of mental health, increased help-seeking among females, changes in diagnostic practices, or changes in outpatient support services. Beyond the ED, there has been a rise in hospitalizations for multiple mental health conditions in female adolescents.^17^ Literature has demonstrated that females consistently reported higher incidence and increases of mental health problems in the COVID-19–prevalent period.^18,19,20^ Hypotheses of these pandemic sex disparities include work disruptions, child responsibilities, and home violence that may have disproportionately affected women,^21^ although the factors may not apply to those 12 to 17 years of age. Young females may have increased vulnerability to stress, school, and extracurricular closures, as well as health service disruptions.^21^ Two large recent systematic reviews suggested that female rates of anxiety and general mental health worsened, albeit by small amounts, with high heterogeneity between studies.^9,22^ Furthermore, biological susceptibility and sociodevelopmental factors associated with peer interactions during crucial periods of adolescence may further explain these sex disparities.^18,19,23^

A previous review suggested that pandemics and their infection control measures are associated with feelings of stress, worry, helplessness, and loneliness in children, which may negatively affect their psychosocial health.^24^ Furthermore, economic burdens including parental unemployment have been associated with adverse mental health outcomes in children,^25^ potentially further compounding the stressors associated with the COVID-19 pandemic.

In many other countries, there was a particularly abrupt rise in admissions for eating disorders in pediatric centers^26,27,28,29,30^ and increased patterns for self-harm^10,31,32^ and suicidality in young females,^33,34^ which parallel our findings. The aforementioned studies^26,27,31,33,34^ suggest that the rise in mental health visits, found in the present study, are supported, and contributing factors should be explored. While the decrease in hospitalizations for mood disorder, substance use disorders, and other conditions is reassuring, it does not imply the absence of psychological distress in youths and young adults; instead, it highlights the limitations of our methods and data.

Understanding how the pandemic affected youths and young adults is crucial to inform public health policy. Recalling the implementation of rigorous measures aimed at mitigating COVID-19–related harm and safeguarding a vulnerable health system, our understanding of the specific groups most susceptible should allow for strategic planning and targeted service provision in the event of another pandemic. While youths and young adults were impacted by the severity of public health measures, disentangling which measures were associated most with the worsening of some mental health presentations is important to inform public health policy. Youth services geared to females, specifically screening for eating disorders and suicidality, will be important to maintain in future pandemics. These services include community outreach, psychological support, and physician or clinical services. Variability in both the pandemic case incidence and public health stringency across provinces may offer a unique and powerful opportunity to generate quantitative evidence of the indirect impacts of COVID-19 on mental health conditions in Canadian youths and young adults to inform public health policies and decision-making during the ongoing COVID-19 pandemic and future pandemics.

### Strengths and Limitations

This study has strengths, including the use of complete health administrative hospitalization data, allowing for the comparison of high-quality accurate admission data for the whole sample size of youths and young adults in Canada, including those in small centers and remote locations. This provided powerful information to compare rates across provinces and territories and inform provincial health policymakers of mental health trends and future needs.

Limitations include the limited variables on the social determinants of health in administrative data and limited complete national ED data. Coding systems, including the type and number of diagnoses in EDs, were not uniform across centers and provinces, and ED results should be interpreted with caution. The NACRS database provided incomplete data in many provinces and was not mandated in others, giving a nongeneralizable portrait of ED visits in all of Canada and an inability to calculate exact incidence rates. Moreover, the lack of accurate and reliable outpatient health administrative data provided us with only a partial understanding of mental health care utilization, especially during a pandemic when hospitals were being avoided. Indeed, hospitalization for mental health conditions illustrates the most severe presentations of these disorders and is likely only the tip of the iceberg with regard to the pandemic’s impact on youths.

## Conclusions

In this cross-sectional study of Canadian youths and young adults, the COVID-19 pandemic was associated with decreased rates of hospitalizations and ED visits for mood disorders, substance use, and other mental health disorders in both sexes but a significant rise in eating disorders and suicide and self-harm in young females across Canada. Policymakers should specifically focus services to support female youths and young adults during the pandemic recovery period and in future pandemic preparedness.

## Notes

Supplement 1. **eTable 1.** National Ambulatory Care Reporting System (NACRS) Database, Number of Submitting Emergency Department by Province/Territory **eTable 2.** *ICD-10* Codes for Mental Health Diagnostic Categories **eTable 3.** Population Estimates for Canadian Children, Adolescents, and Young Adults Aged 6 to 21 Years Across the Study Period **eTable 4.** Characteristics of Mental Health Hospitalizations by Fiscal Year **eFigure 1.** Rates of Mental Health Hospitalizations by 10 000 Person-Years for Each Diagnostic Subgroup and Each Region, Including Territories **eFigure 2.** Rates of Mental Health Hospitalizations by 10 000 Person-Years by Diagnostic Subgroup by Age Group **eFigure 3.** Rates of Mental Health Hospitalizations by 10 000 Person-Years for Each Diagnostic Subgroup Stratified by Area (Urban/Rural) **eTable 5.** Characteristics of Mental Health Emergency Department Visits Pre–COVID-19 and COVID-19–Prevalent Periods **eTable 6.** Characteristics of Mental Health Emergency Department Visits by Fiscal Year (N = 881,765) **eFigure 4.** Rates of Mental Health Emergency Department Visits by 10 000 Person-Years for Each Diagnostic Subgroup Stratified by Provinces (With Complete ED Reporting) **eTable 7.** Emergency Department Visit Rate by 10 000 Person-Years, for Mental Health Disorders Pre–COVID-19 Period vs COVID-19–Prevalent Period (Ontario, Quebec, Alberta, and Yukon) by Sex

Supplement 2. **Nonauthor Collaborators.** The POPCORN Investigators

Supplement 3.
Data Sharing Statement

## References

[1] Ludvigsson JF. Systematic review of COVID-19 in children shows milder cases and a better prognosis than adults. Acta Paediatr. 2020;109(6):1088-1095. doi:10.1111/apa.15270 32202343 PMC7228328

[2] Hillis SD, Unwin HJT, Chen Y, . Global minimum estimates of children affected by COVID-19-associated orphanhood and deaths of caregivers: a modelling study. Lancet. 2021;398(10298):391-402. doi:10.1016/S0140-6736(21)01253-8 34298000 PMC8293949

[3] Thompson J, Spencer G, Curtis P. Children’s perspectives and experiences of the COVID-19 pandemic and UK public health measures. Health Expect. 2021;24(6):2057-2064. doi:10.1111/hex.13350 34495568 PMC8628584

[4] Fore HH, Hijazi Z. COVID-19 is hurting children’s mental health. here are 3 ways we can help. World Economic Forum. May 1, 2020. Accessed January 15, 2024. https://www.weforum.org/agenda/2020/05/covid-19-is-hurting-childrens-mental-health/

[5] Cameron-Blake E, Annan H, Marro L, Michaud D, Sawatzky J, Tatlow H. Variation in the stringency of COVID-19 public health measures on self-reported health, stress, and overall wellbeing in Canada. Sci Rep. 2023;13(1):13094. doi:10.1038/s41598-023-39004-w 37567870 PMC10421886

[6] Dyer O. Covid-19: Canada outperformed comparable nations in pandemic response, study reports. BMJ. 2022;377:o1615. doi:10.1136/bmj.o1615 35772776

[7] Razak F, Shin S, Naylor CD, Slutsky AS. Canada’s response to the initial 2 years of the COVID-19 pandemic: a comparison with peer countries. CMAJ. 2022;194(25):E870-E877. doi:10.1503/cmaj.220316 35760433 PMC9332918

[8] UNICEF. The impact of COVID-19 on the mental health of adolescents and youth. 2020. Accessed January 11, 2024. https://www.unicef.org/lac/en/impact-covid-19-mental-health-adolescents-and-youth

[9] Madigan S, Racine N, Vaillancourt T, . Changes in depression and anxiety among children and adolescents from before to during the COVID-19 pandemic: a systematic review and meta-analysis. JAMA Pediatr. 2023;177(6):567-581. doi:10.1001/jamapediatrics.2023.0846 37126337 PMC10152379

[10] Madigan S, Korczak DJ, Vaillancourt T, . Comparison of paediatric emergency department visits for attempted suicide, self-harm, and suicidal ideation before and during the COVID-19 pandemic: a systematic review and meta-analysis. Lancet Psychiatry. 2023;10(5):342-351. doi:10.1016/S2215-0366(23)00036-6 36907199 PMC10097509

[11] Gutiérrez-Sacristán A, Serret-Larmande A, Hutch MR, ; Consortium for Clinical Characterization of COVID-19 by EHR (4CE). Hospitalizations associated with mental health conditions among adolescents in the US and France during the COVID-19 pandemic. JAMA Netw Open. 2022;5(12):e2246548. doi:10.1001/jamanetworkopen.2022.46548 36512353 PMC9856226

[12] Karmakar M, Lantz PM, Tipirneni R. Association of social and demographic factors with COVID-19 incidence and death rates in the US. JAMA Netw Open. 2021;4(1):e2036462. doi:10.1001/jamanetworkopen.2020.36462 33512520 PMC7846939

[13] Abrams EM, Greenhawt M, Shaker M, Pinto AD, Sinha I, Singer A. The COVID-19 pandemic: adverse effects on the social determinants of health in children and families. Ann Allergy Asthma Immunol. 2022;128(1):19-25. doi:10.1016/j.anai.2021.10.022 34699969 PMC8539831

[14] Altman DG, Simera I, Hoey J, Moher D, Schulz K. EQUATOR: reporting guidelines for health research. Lancet. 2008;371(9619):1149-1150. doi:10.1016/S0140-6736(08)60505-X 18395566

[15] R. The R Project for Statistical Computing; 2021. Accessed March 20, 2024. https://www.R-project.org/

[16] Kurdyak P, Gandhi S, Holder L, . Incidence of access to ambulatory mental health care prior to a psychiatric emergency department visit among adults in Ontario, 2010-2018. JAMA Netw Open. 2021;4(4):e215902. doi:10.1001/jamanetworkopen.2021.5902 33852001 PMC8047734

[17] Saunders NR, Kurdyak P, Stukel TA, . Utilization of physician-based mental health care services among children and adolescents before and during the COVID-19 pandemic in Ontario, Canada. JAMA Pediatr. 2022;176(4):e216298. doi:10.1001/jamapediatrics.2021.6298 35129604 PMC8822447

[18] Penninx BWJH, Benros ME, Klein RS, Vinkers CH. How COVID-19 shaped mental health: from infection to pandemic effects. Nat Med. 2022;28(10):2027-2037. doi:10.1038/s41591-022-02028-2 36192553 PMC9711928

[19] Racine N, McArthur BA, Cooke JE, Eirich R, Zhu J, Madigan S. Global prevalence of depressive and anxiety symptoms in children and adolescents during COVID-19: a meta-analysis. JAMA Pediatr. 2021;175(11):1142-1150. doi:10.1001/jamapediatrics.2021.2482 34369987 PMC8353576

[20] COVID-19 Mental Disorders Collaborators. Global prevalence and burden of depressive and anxiety disorders in 204 countries and territories in 2020 due to the COVID-19 pandemic. Lancet. 2021;398(10312):1700-1712. doi:10.1016/S0140-6736(21)02143-7 34634250 PMC8500697

[21] Flor LS, Friedman J, Spencer CN, . Quantifying the effects of the COVID-19 pandemic on gender equality on health, social, and economic indicators: a comprehensive review of data from March, 2020, to September, 2021. Lancet. 2022;399(10344):2381-2397. doi:10.1016/S0140-6736(22)00008-3 35247311 PMC8890763

[22] Sun Y, Wu Y, Fan S, . Comparison of mental health symptoms before and during the covid-19 pandemic: evidence from a systematic review and meta-analysis of 134 cohorts. BMJ. 2023;380:e074224. doi:10.1136/bmj-2022-074224 36889797 PMC9992728

[23] Riecher-Rössler A. Sex and gender differences in mental disorders. Lancet Psychiatry. 2017;4(1):8-9. doi:10.1016/S2215-0366(16)30348-0 27856397

[24] Meherali S, Punjani N, Louie-Poon S, . Mental health of children and adolescents amidst COVID-19 and past pandemics: a rapid systematic review. Int J Environ Res Public Health. 2021;18(7):3432. doi:10.3390/ijerph18073432 33810225 PMC8038056

[25] Golberstein E, Gonzales G, Meara E. How do economic downturns affect the mental health of children? evidence from the National Health Interview Survey. Health Econ. 2019;28(8):955-970. doi:10.1002/hec.3885 31165566 PMC7427110

[26] Agostino H, Burstein B, Moubayed D, . Trends in the incidence of new-onset anorexia nervosa and atypical anorexia nervosa among youth during the COVID-19 pandemic in Canada. JAMA Netw Open. 2021;4(12):e2137395. doi:10.1001/jamanetworkopen.2021.37395 34874405 PMC8652595

[27] Toulany A, Kurdyak P, Guttmann A, . Acute care visits for eating disorders among children and adolescents after the onset of the COVID-19 pandemic. J Adolesc Health. 2022;70(1):42-47. doi:10.1016/j.jadohealth.2021.09.025 34690054 PMC8530790

[28] Asch DA, Buresh J, Allison KC, . Trends in US patients receiving care for eating disorders and other common behavioral health conditions before and during the COVID-19 pandemic. JAMA Netw Open. 2021;4(11):e2134913. doi:10.1001/jamanetworkopen.2021.34913 34783829 PMC8596194

[29] Toulany A, Saunders NR, Kurdyak P, . Acute presentations of eating disorders among adolescents and adults before and during the COVID-19 pandemic in Ontario, Canada. CMAJ. 2023;195(38):E1291-E1299. doi:10.1503/cmaj.221318 37788846 PMC10637328

[30] J Devoe D, Han A, Anderson A, . The impact of the COVID-19 pandemic on eating disorders: a systematic review. Int J Eat Disord. 2023;56(1):5-25. doi:10.1002/eat.23704 35384016 PMC9087369

[31] Cousien A, Acquaviva E, Kernéis S, Yazdanpanah Y, Delorme R. Temporal trends in suicide attempts among children in the decade before and during the COVID-19 pandemic in Paris, France. JAMA Netw Open. 2021;4(10):e2128611. doi:10.1001/jamanetworkopen.2021.28611 34618041 PMC8498848

[32] Hill RM, Rufino K, Kurian S, Saxena J, Saxena K, Williams L. Suicide ideation and attempts in a pediatric emergency department before and during COVID-19. Pediatrics. 2021;147(3):e2020029280. doi:10.1542/peds.2020-029280 33328339

[33] Ray JG, Austin PC, Aflaki K, Guttmann A, Park AL. Comparison of self-harm or overdose among adolescents and young adults before vs during the COVID-19 pandemic in Ontario. JAMA Netw Open. 2022;5(1):e2143144. doi:10.1001/jamanetworkopen.2021.43144 35019981 PMC8756304

[34] Poonai N, Freedman SB, Newton AS, ; Pediatric Emergency Research Canada (PERC) Network. Emergency department visits and hospital admissions for suicidal ideation, self-poisoning and self-harm among adolescents in Canada during the COVID-19 pandemic. CMAJ. 2023;195(36):E1221-E1230. doi:10.1503/cmaj.220507 37722746 PMC10506508

