# Supplementary material for: Mental Health Hospitalizations in Canadian Children, Adolescents, and Young Adults Over the COVID-19 Pandemic
Source: JAMA Netw Open. 2024 Jul 8;7(7):e2422833. doi: 10.1001/jamanetworkopen.2024.22833 (PMC11231797; doi:10.1001/jamanetworkopen.2024.22833)
Supplement: Supplement 1. — eTable 1. National Ambulatory Care Reporting System (NACRS) Database, Number of Submitting Emergency Department by Province/Territory eTable 2. ICD-10 Codes for Mental Health Diagnostic Categories eTable 3. Population Estimates for Canadian Children, Adolescents, and Young Adults Aged 6 to 21 Years Across the Study Period eTable 4. Characteristics of Mental Health Hospitalizations by Fiscal Year eFigure 1. Rates of Mental Health Hospitalizations by 10 000 Person-Years for Each Diagnostic Subgroup and Each Region, Including Territories eFigure 2. Rates of Mental Health Hospitalizations by 10 000 Person-Years by Diagnostic Subgroup by Age Group eFigure 3. Rates of Mental Health Hospitalizations by 10 000 Person-Years for Each Diagnostic Subgroup Stratified by Area (Urban/Rural) eTable 5. Characteristics of Mental Health Emergency Department Visits Pre–COVID-19 and COVID-19–Prevalent Periods eTable 6. Characteristics of Mental Health Emergency Department Visits by Fiscal Year (N = 881,765) eFigure 4. Rates of Mental Health Emergency Department Visits by 10 000 Person-Years for Each Diagnostic Subgroup Stratified by Provinces (With Complete ED Reporting) eTable 7. Emergency Department Visit Rate by 10 000 Person-Years, for Mental Health Disorders Pre–COVID-19 Period vs COVID-19–Prevalent Period (Ontario, Quebec, Alberta, and Yukon) by Sex [file jamanetwopen-e2422833-s001.pdf]

Supplemental Online Content

Roumeliotis N, Carwana M, Trudeau O, et al. Mental health hospitalizations in Canadian children, adolescents, and young adults over the COVID-19 pandemic. *JAMA Netw Open*. 2024;7(7):e2422833. doi:10.1001/jamanetworkopen.2024.22833

eTable 1. National Ambulatory Care Reporting System (NACRS) Database, Number of Submitting Emergency Department by Province/Territory
eTable 2. *ICD-10* Codes for Mental Health Diagnostic Categories
eTable 3. Population Estimates for Canadian Children, Adolescents, and Young Adults Aged 6 to 21 Years Across the Study Period
eTable 4. Characteristics of Mental Health Hospitalizations by Fiscal Year
eFigure 1. Rates of Mental Health Hospitalizations by 10 000 Person-Years for Each Diagnostic Subgroup and Each Region, Including Territories
eFigure 2. Rates of Mental Health Hospitalizations by 10 000 Person-Years by Diagnostic Subgroup by Age Group
eFigure 3. Rates of Mental Health Hospitalizations by 10 000 Person-Years for Each Diagnostic Subgroup Stratified by Area (Urban/Rural)
eTable 5. Characteristics of Mental Health Emergency Department Visits Pre–COVID-19 and COVID-19–Prevalent Periods
eTable 6. Characteristics of Mental Health Emergency Department Visits by Fiscal Year (N = 881,765)
eFigure 4. Rates of Mental Health Emergency Department Visits by 10 000 Person-Years for Each Diagnostic Subgroup Stratified by Provinces (With Complete ED Reporting)
eTable 7. Emergency Department Visit Rate by 10 000 Person-Years, for Mental Health Disorders Pre–COVID-19 Period vs COVID-19–Prevalent Period (Ontario, Quebec, Alberta, and Yukon) by Sex

This supplemental material has been provided by the authors to give readers additional information about their work.

© 2024 Roumeliotis N et al. *JAMA Network Open*.

eTable 1. National Ambulatory Care Reporting System (NACRS) Database, Number of Submitting Emergency Department by Province/Territory

Province / territory	No. of submitting emergency department
Prince Edward Island	1
Nova Scotia	4
Newfoundland and Labrador	0
New Brunswick	0
Ontario	179
Manitoba	0
Saskatchewan	10
Alberta	109
British Columbia	29
Yukon	3
Northwest Territories and Nunavut	0
Total	335

Data are complete for Ontario, Alberta, Yukon from CIHI.
Data are complete from Quebec from INESSS.

© 2024 Roumeliotis N et al. *JAMA Network Open*.

eTable 2. *ICD-10* Codes for Mental Health Diagnostic Categories

Mental/Behavioural disorder group	ICD-10 codes (range)
Suicide and self-harm	X60-84
Mood Disorders	F30 - F33, F39, F340, F341, F348, F349, F380, F381, F388
Schizophrenic/Psychotic Disorders	F20 - F29
Anxiety Disorders	F40-F42, F430, F431, F438, F439, F930 - F932
Personality Disorders	F60 - F62, F68, F69
Eating Disorders	F50
Substance -related Disorders	F10 - F19, F55
Other mental/Behavioural Disorders: Adjustment disorders (culture shock, grief reaction, hospitalism in children); dissociative disorders, specific developmental disorders of speech and language; specific developmental disorder of motor function; behavioral/emotional disorders with onset usually in childhood and adolescence including TIC disorder, Autism, ADHD	All other mental health codes (F10-F99) not included in previous categories.<br><br>F43, F44, F45, F48, F59, F53, F54, F63 -65, F66, F70-73, F78 - F89, F90, F91, F92, F933 - F939, F94, F95, F99

ICD-10-CA = International Statistical Classification of Diseases and Related Health Problems,10th Revision, Canada.
Personality disorder diagnosis is restricted to patients age 18-20 years.

© 2024 Roumeliotis N et al. *JAMA Network Open*.

eTable 3. Population Estimates for Canadian Children, Adolescents, and Young Adults Aged 6 to 21 Years Across the Study Period

	Fiscal Year						
**Characteristic**	**2016-17**	**2017-18**	**2018-19**	**2019-20**	**2020-21**	**2021-22**	**2022-23**
**Total**	6 076 121	6 123 912	6 202 774	6 269 212	6 304 676	6 261 795	6 390 438
**Age group**							
[6-12)	2 376 965 (39.1)	2 412 801(39.4)	2 449 067 (39.5)	2 474 268 (39.5)	2 483 683 (39.4)	2 477 877 (39.6)	2 504 141 (39.2)
[12-18)	2 365 592 (38.9)	2 369 205 (38.7)	2 379 187 (38.4)	2 406 949 (38.4)	2 444 547 (38.8)	2 468 893 (39.4)	2 528 132 (39.6)
[18-21)	1 333 564 (21.9)	1 341 906 (21.9)	1 374 520 (22.2)	1 387 995 (22.1)	1 376 446 (21.8)	1 315 025 (21.0)	1 358 165 (21.3)
**Sex**							
Male	3 112 552 (51.2)	3 136 107 (51.2)	3 174 420 (51.2)	3 207 157 (51.2)	3 223 202 (51,1)	3 198 994 (51.1)	3 264 647 (51.1)
Female	2 963 569 (48.8)	2 987 805 (48.8)	3 028 354 (48.8)	3 062 055 (48.8)	3 081 474 (48,9)	3 062 801 (48.9)	3 125 791 (48.9)
**Area**							
Urban	5 058 041 (83.2)	5 101 149 (83.3)	5 174 741 (83.5)	5 236 939 (83.5)	5 269 412 (83.6)	5 222 201 (83.4)	5 334 041 (83.5)
Rural	1 018 080 (16.8)	1 022 763 (16.7)	1 028 033 (16.6)	1 032 273 (16.5)	1 035 264 (16.4)	1 039 594 (16.6)	1 056 397 (16.5)
**Material deprivation[a]**							
1 (least deprived)	1 161 730 (19.2)	1 170 846 (19.2)	1 185 920 (19.2)	1 198 617 (19.2)	1 205 318 (19.2)	1 197 023 (19.2)	1 221 661 (19.2)
2	1 228 287 (20.3)	1 237 926 (20.3)	1 253 863 (20.3)	1 267 288 (20.3)	1 274 372 (20.3)	1 265 603 (20.3)	1 291 652 (20.3)
3	1 204 085 (19.9)	1 213 533 (19.9)	1 229 157 (19.9)	1 242317 (19.9)	1 249 262 (19.9)	1 240 665 (19.9)	1 266 201 (19.9)
4	1 179 882 (19.5)	1 189 140 (19.5)	1 204 450 (19.5)	1 217 345 (19.5)	1 224 151 (19.5)	1 215 727 (19.5)	1 240 750 (19.5)
5 (most deprived)	1 276 693 (21.1)	1 286 711 (21.1)	1 303 277 (21.1)	1 317 230 (21.1)	1 3245 94 (21.1)	1 315 479 (21.1)	1 342 555 (21.1)
**Provinces/Territories**							
Prince Edward Island	25 130 (0.4)	25 649 (0.4)	26 197 (0.4)	26 923 (0.4)	27 375 (0.4)	27 143 (0.4)	27 733 (0.4)
New Brunswick	118 559 (2.0)	118 689 (1.9)	119 277 (1.9)	12 0192 (1.9)	120 882 (1.9)	120 486 (1.9)	124 175 (1.9)
Newfoundland and Labrador	80 834 (1.3)	80 378 (1.3)	79 556 (1.3)	78 688 (1.3)	77 997 (1.2)	76 720 (1.2)	77 683 (1.2)
Nova Scotia	146 620 (2.4)	146 599 (2.4)	147 034 (2.4)	14 7878 (2.4)	148 409 (2.4)	146 787 (2.3)	150 847 (2.4)
Québec	1 287 873 (21.2)	1 296 080 (21.2)	1 313 282 (21.2)	1 335 353 (21.3)	1 356 037 (21.5)	1 357 072 (21.7)	1 375 050 (21.5)
Ontario	2 402 088 (39.5)	2 417 569 (39.5)	2 447 055 (39.5)	2 462 779 (39.3)	2 463 205 (39.1)	2 427 970 (38.8)	2 473 364 (38.7)
Manitoba	252 362 (4.2)	255 843 (4.2)	259 606(4.2)	263 178 (4.2)	264 408 (4.2)	263 941 (4.2)	267 495 (4.2)
Saskatchewan	213 677 (3.5)	216 959 (3.5)	219 947 (3.5)	223 553 (3.6)	226 076 (3.6)	226 952 (3.6)	231 536 (3.6)

© 2024 Roumeliotis N et al. *JAMA Network Open*.

	2016-17	2017-18	2018-19	2019-20	2020-21	2021-22	2022-23
Alberta	762 460 (12.5)	772 576 (12.6)	785 932 (12.7)	798 805 (12.7)	808 772 (12.8)	812 930 (13.0)	837 740 (13.1)
British Columbia	761 074 (12.5)	767 814 (12.5)	778 780 (12.6)	785 448 (12.5)	784 535 (12.4)	774 495 (12.4)	797 195 (12.5)
Yukon	6256 (0.1)	6 425 (0.1)	6 575 (0.1)	6 653 (0.1)	6 849 (0.1)	7 007 (0.1)	7 158 (0.1)
Northwest Territories	8694 (0.1)	8 661 (0.1)	8 693 (0.1)	8 724 (0.1)	8 822 (0.1)	8 848 (0.1)	8 853 (0.1)
Nunavut	10 494 (0.2)	10 670 (0.2)	10 840 (0.2)	11 038 (0.2)	11 309 (0.2)	11444 (0.2)	11 609 (0.2)

All numbers are expressed a N (%) of column total.

a. Quintile Rankings: Lower scores correspond to areas least marginalized, while higher scores for each dimension relate to areas most marginalized.

© 2024 Roumeliotis N et al. *JAMA Network Open*.

eTable 4. Characteristics of Mental Health Hospitalizations by Fiscal Year

	Fiscal Year						
**Characteristic**	**2016 - 2017**	**2017 - 2018**	**2018 - 2019**	**2019 - 2020**	**2020 - 2021**	**2021 - 2022**	**2022 - 2023**
Total	30 694	33 246	32 598	30 735	29 666	33567	27 595
Sex[a], n (%)							
Female	19735 (64.3)	21216 (63.9)	20748 (63.7)	19476 (63.4)	20 004 (67.5)	23 592 (70.4)	18 928 (68.8)
Male	10941 (35.7)	11990 (36.1)	11816 (36.3)	11241 (36.6)	9620 (32.5)	9904 (29.6)	8587 (31.2)
Age group, n (%)							
6-11	1822 (5.9)	2008 (6.0)	2084 (6.4)	1993 (6.5)	1632 (5.5)	1779 (5.3)	1424 (5.2)
12-17	20254 (66.0)	21945 (66.0)	21408 (65.7)	20113 (65.4)	19 808 (66.8)	23272 (69.3)	19 006 (68.9)
18-20	8618 (28.1)	9293 (28.0)	9106 (27.9)	8629 (27.9)	8226 (27.7)	8516 (25.4)	7165 (26.0)
Rural address[b], n (%)	6401 (21.6)	6940 (21.7)	6514 (20.9)	6138 (20.7)	5635 (19.6)	6290 (19.4)	5443 (20.2)
Material deprivation quintile[c]							
1 (less deprived)	4736 (16.6)	5229 (17.0)	5032 (16.8)	4946 (17.3)	5301 (19.2)	5921 (19.0)	4116 (17.3)
2	5593 (19.5)	5992 (19.4)	5954 (19.9)	5595 (19.6)	5571 (120.2)	6389 (20.6)	4611 (19.4)
3	5601 (19.6)	5978 (19.4)	5815 (19.4)	5511 (19.3)	5406 (19.6)	6167 (19.8)	4833 (20.3)
4	5719 (20.0)	6056 (19.6)	5843 (19.5)	5627 (19.7)	5364 (19.4)	5924 (19.1)	4844 (20.4)
5 (most deprived)	6961 (24.3)	7576 (24.6)	7325 (24.4)	6908 (24.2)	5986 (21.7)	6685 (21.5)	5370 (22.6)
Social deprivation quintile[c]							
1 (less deprived)	5726 (20.0)	6431 (20.8)	6179 (20.6)	5807 (20.3)	5741 (20.8)	6478 (20.8)	4478 (18.8)
2	6002 (21.0)	6152 (19.9)	6003 (20.0)	5745 (20.1)	5679 (20.5)	6531 (21.0)	4563 (19.2)
3	5593 (19.5)	6161 (20.0)	5900 (19.7)	5559 (19.4)	5628 (20.3)	6172 (19.8)	4609 (19.4)
4	5548 (19.4)	5969 (19.3)	5966 (19.9)	5688 (19.9)	5408 (19.6)	6163 (19.8)	5041 (21.2)
5 (most deprived)	5780 (20.2)	6163 (20.0)	5966 (19.9)	5821 (20.3)	5202 (18.8)	5769 (18.5)	5117 (21.5)
Province/Territories							
Prince Edward Island	276 (0.9)	261 (0.8)	270 (0.8)	247 (0.8)	110 (0.4)	195 (0.6)	109 (0.4)
New Brunswick	929 (3.0)	1016 (3.1)	1001 (3.1)	845 (2.7)	893 (3.0)	990 (2.9)	748 (2.7)
Newfoundland and Labrador	485 (1.6)	527 (1.6)	515 (1.6)	399 (1.3)	367 (1.2)	352 (1.0)	254 (0.9)
Nova Scotia	644 (2.1)	577 (1.7)	498 (1.5)	518 (1.7)	472 (1.6)	520 (1.5)	366 (1.3)
Québec	5908 (19.2)	6468 (19.5)	6387 (19.6)	5965 (19.4)	5635 (19.0)	6480 (19.3)	5732 (20.8)
Ontario	10098 (32.9)	10696 (32.2)	10481 (32.2)	9652 (31.4)	9555 (32.2)	10804 (32.2)	9710 (35.2)

© 2024 Roumeliotis N et al. *JAMA Network Open*.

	2016 - 2017	2017 - 2018	2018 - 2019	2019 - 2020	2020 - 2021	2021 - 2022	2022 - 2023
Manitoba	1123 (3.7)	1096 (3.3)	1122 (3.4)	1041 (3.4)	905 (3.1)	1066 (3.2)	846 (3.1)
Saskatchewan	2188 (7.1)	2352 (7.1)	2275 (7.0)	2045 (6.7)	1688 (5.7)	1964 (5.9)	1803 (6.5)
Alberta	3932 (12.8)	4194 (12.6)	4116 (12.6)	4012 (13.1)	4198 (14.2)	4607 (13.7)	3869 (14.0)
British Columbia	4891 (15.9)	5766 (17.3)	5546 (17.0)	5696 (18.5)	5533 (18.7)	6245 (18.6)	3937 (14.3)
Territories	220 (0.7)	293 (0.9)	387 (1.2)	315 (1.0)	310 (1.0)	344 (1.0)	221 (0.8)
Diagnostic subgroup, n (%)							
Anxiety	3498 (11.4)	3920 (11.8)	4026 (12.4)	3981 (13.0)	4018 (13.5)	4651 (13.9)	3686 (13.4)
Eating disorders	1200 (3.9)	1192 (3.6)	1298 (4.0)	1294 (4.2)	2082 (7.0)	2347 (7.0)	1876 (6.8)
Mood disorders	7517 (24.5)	8329 (25.1)	7917 (24.3)	7244 (23.6)	6708 (22.6)	7684 (22.9)	5686 (20.6)
Other mental/behavioral disorders	8457 (27.6)	9086 (27.3)	8708 (26.7)	8241 (26.8)	6642 (22.4)	7633 (22.7)	6360 (23.0)
Personality disorders	1601 (5.2)	1732 (5.2)	1835 (5.6)	1734 (5.6)	1750 (5.9)	2252 (6.7)	2131 (7.7)
Schizophrenic/Psychotic	2191 (7.1)	2161 (6.5)	2133 (6.5)	2085 (6.8)	2152 (7.3)	2186 (6.5)	1853 (6.7)
Substance related disorders	2475 (8.1)	2604 (7.8)	2580 (7.9)	2463 (8.0)	2418 (8.2)	2153 (6.4)	1900 (6.9)
Suicide and self-harm	3755 (12.2)	4222 (12.7)	4101 (12.6)	3693 (12.0)	3896 (13.1)	4661 (13.9)	4103 (14.9)
Transfer between centers, n (%)	3840 (12.5)	3869 (11.6)	4504 (13.8)	4051 (13.2)	4467 (15.1)	5169 (15.4)	4355 (15.8)
Pediatric center[d], n (%)							
Yes	6697 (21.8)	6960 (20.9)	6981 (21.4)	6557 (21.3)	7319 (24.7)	8514 (25.4)	6848 (24.8)
No	23997 (78.2)	26286 (79.1)	25617 (78.6)	24178 (78.7)	22347 (75.3)	25053 (74.6)	20747 (75.2)
Mental health visit in last 2 years, n (%)							
None	14208 (46.3)	15138 (45.5)	14400 (44.2)	13671 (44.5)	13280 (44.8)	15056 (44.9)	11635 (42.2)
Emergency Department visit	8860 (28.9)	9865 (29.7)	9922 (30.4)	9310 (30.3)	8933 (25.1)	10338 (24.4)	9091 (24.9)
Hospitalization	7626 (24.8)	8243 (24.8)	8276 (25.4)	7754 (25.2)	7453 (30.1)	8173 (30.8)	6869 (32.9)

a. Sex other n=303 (0.1%), not shown
b. Missing Urban/rural designation n=7413 (3.4%)
c. Missing Material and Social deprivation for n= 17 616 (8.1%) and n=17 363 (8.0%) respectively.
d. Defined as a tertiary center with a pediatric intensive care unit

© 2024 Roumeliotis N et al. *JAMA Network Open*.

eFigure 1. Rates of Mental Health Hospitalizations by 10 000 Person-Years for Each Diagnostic Subgroup and Each Region, Including Territories

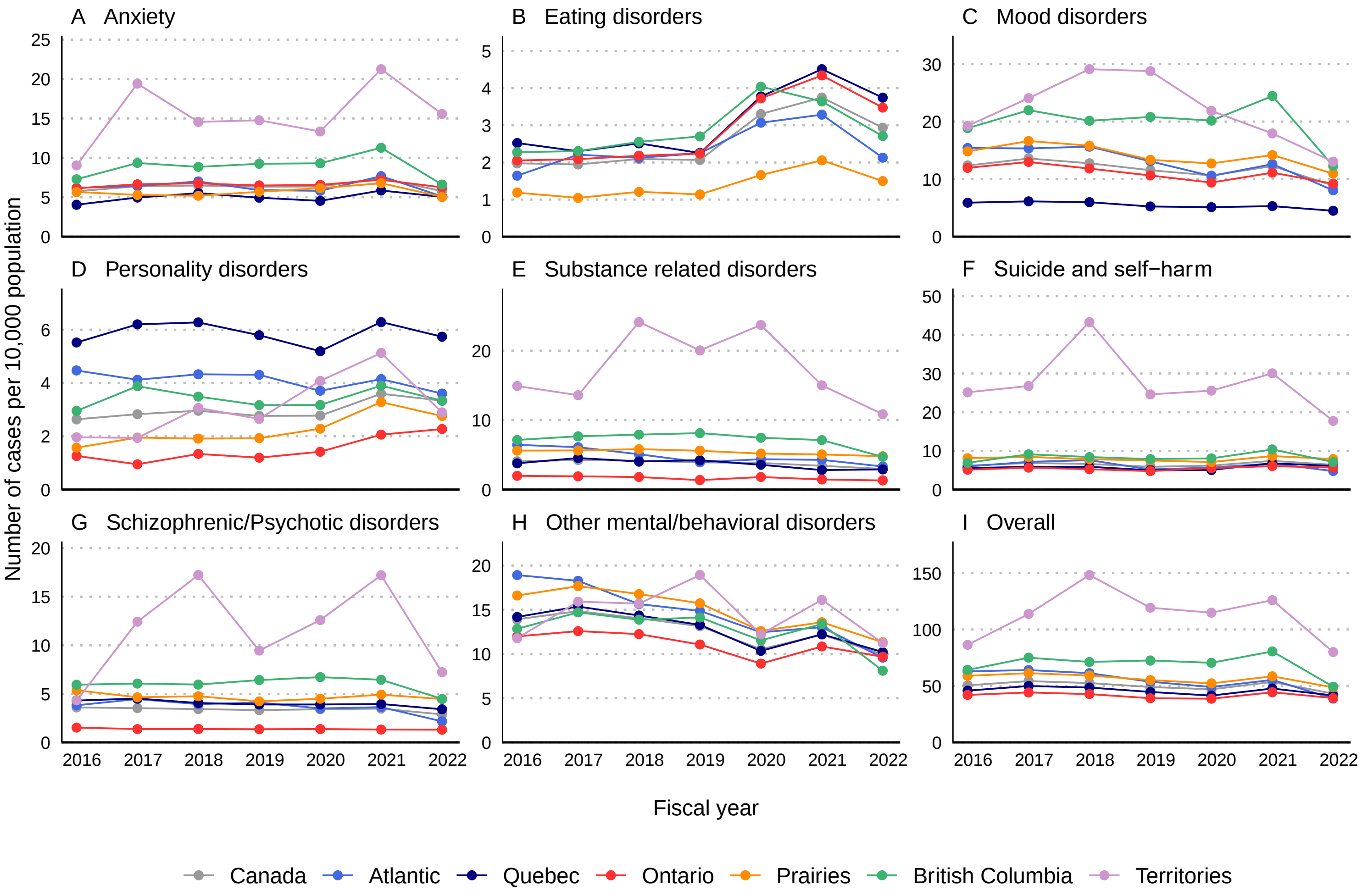

Points correspond to aggregate data by fiscal year, and are displayed at the beginning of the fiscal year.

© 2024 Roumeliotis N et al. *JAMA Network Open*.

eFigure 2. Rates of Mental Health Hospitalizations by 10 000 Person-Years by Diagnostic Subgroup by Age Group

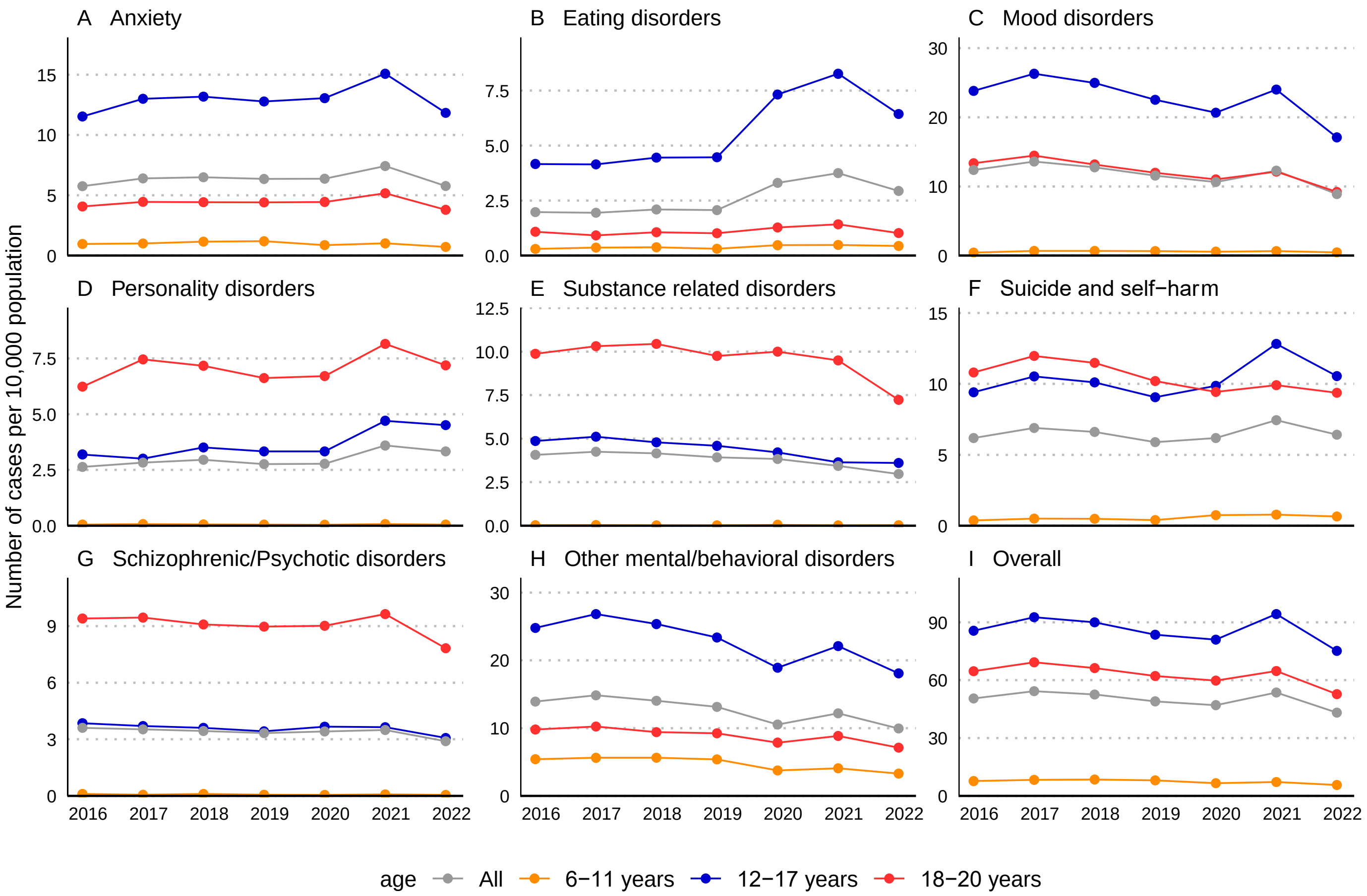

Points correspond to aggregate data by fiscal year, and are displayed at the beginning of the fiscal year.

© 2024 Roumeliotis N et al. *JAMA Network Open*.

eFigure 3. Rates of Mental Health Hospitalizations by 10 000 Person-Years for Each Diagnostic Subgroup Stratified by Area (Urban/Rural)

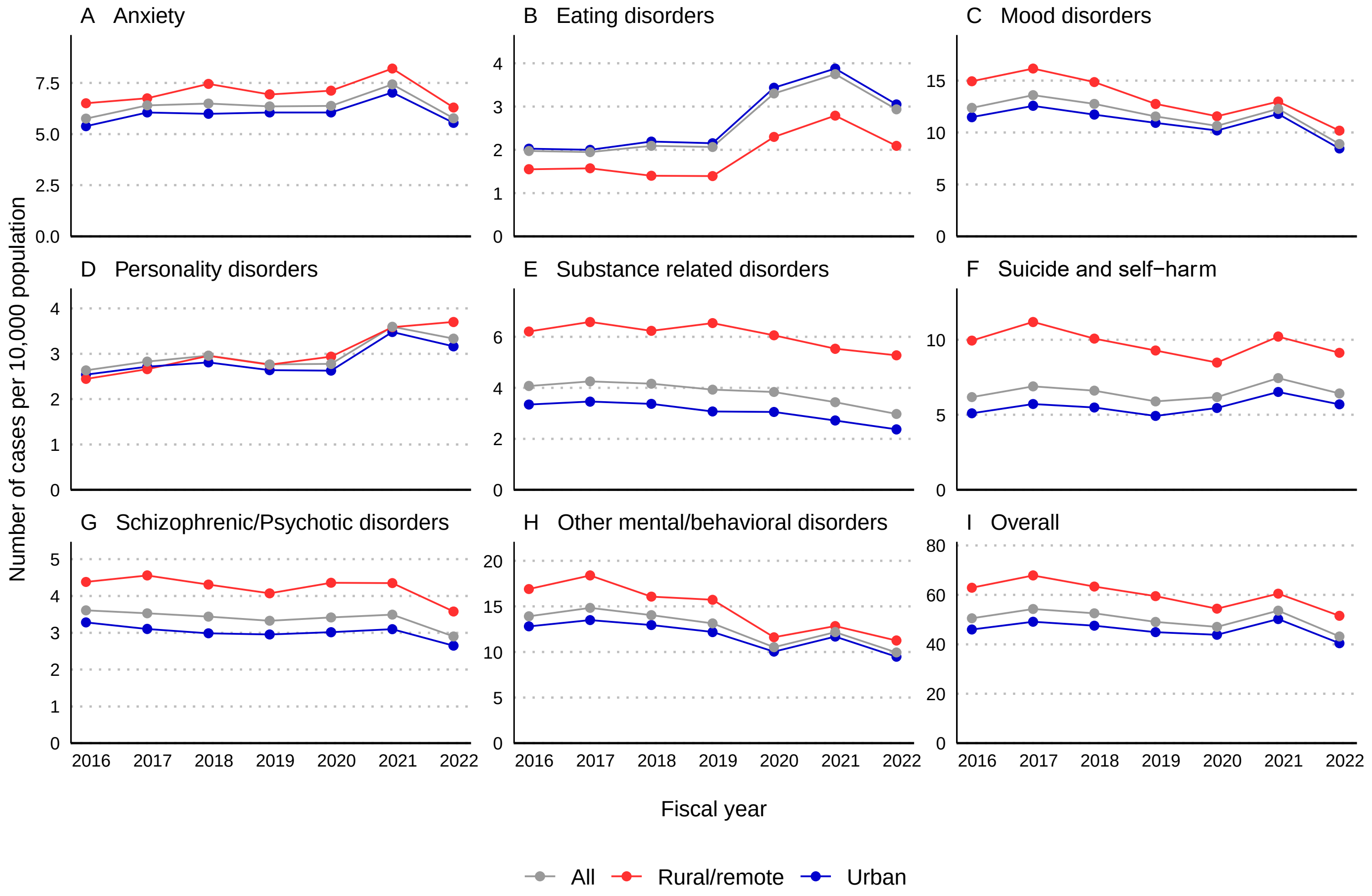

Points correspond to aggregate data by fiscal year, and are displayed at the beginning of the fiscal year.

© 2024 Roumeliotis N et al. *JAMA Network Open*.

eTable 5. Characteristics of Mental Health Emergency Department Visits Pre–COVID-19 and COVID-19–Prevalent Periods

Characteristic	Overall (N = 881 765)	Pre-COVID (n = 528 280)	COVID-prevalent (n = 353 485)	*P* value
Sex[a], n (%)				
Female	549 100 (62.3)	316 953 (60.0)	232 147 (65.8)	**<.001**
Male	331 867 (37.7)	210 974 (40.0)	120 893 (34.2)	
Age group, n (%)				
6-11 years	61 676 (7.0)	36 703 (6.9)	24 973 (7.1)	**<.001**
12-17 years	429 635 (48.7)	243 272 (46.0)	186 363 (52.7)	
18-20 years	390 454 (44.3)	248 305 (47.0)	142 149 (40.2)	
Rural address[b], n (%)	138 220 (16.8)	81 267 (16.7)	56 953 (17.0)	**0.007**
Material deprivation quintile[c], n (%)				
1 (less deprived)	151 622 (21.2)	88 851 (21.3)	62 771 (21.0)	**<.001**
2	151 043 (19.1)	90 559 (19.0)	60 484 (19.2)	
3	150 629 (18.5)	89 170 (18.4)	61 459 (18.7)	
4	151 287 (19.8)	90 417 (19.7)	60 870 (20.0)	
5 (most deprived)	179 116 (21.3)	109 210 (21.5)	69 906 (21.1)	
Province/Territories[d], n (%)				
Prince Edward Island	1 996 (0.2)	1 088 (0.2)	908 (0.3)	**<.001**
Nova Scotia	10 372 (1.2)	5 870 (1.1)	4 502 (1.3)	
Quebec	266 919 (30.3)	161 306 (30.5)	105 613 (29.9)	
Ontario	378 546 (42.9)	231 464 (43.8)	147 082 (41.6)	
Saskatchewan	31 901 (3.6)	17 294 (3.3)	14 607 (4.1)	
Alberta	141 879 (16.1)	82 579 (15.6)	59 300 (16.8)	
British Columbia	47 908 (5.4)	27 382 (5.2)	20 526 (5.8)	
Yukon	2 244 (0.3)	1 297 (0.2)	947 (0.3)	
Diagnostic subgroup, n (%)				
Anxiety	237 773 (27.0)	138 135 (26.1)	99 638 (28.2)	**<.001**
Eating disorders	8 197 (0.9)	3 088 (0.6)	5 109 (1.4)	
Mood disorders	162 745 (18.5)	99 543 (18.8)	63 202 (17.9)	
Other mental disorders	158 533 (18.0)	91 954 (17.4)	66 579 (18.8)	
Personality disorders	36 975 (4.2)	22 164 (4.2)	14 811 (4.2)	
Schizophrenic/Psychotic	30 473 (3.5)	18 056 (3.4)	12 417 (3.4)	
Substance related disorders	139 719 (15.8)	95 615 (18.1)	44 104 (18.1)	
Suicide and self-harm[e]	107 350 (12.2)	59 725 (11.3)	47 625 (11.3)	
Transfer between centers, n (%)	177 125 (20.1)	101 688 (19.2)	75 437 (21.3)	**<.001**
Admitted in pediatric center[f], n (%)				
Yes	167 682 (19.0)	84 556 (16.0)	83 126 (23.5)	**<.001**
No	714083 (81.0)	443724 (84.0)	270359 (76.5)	
Mental health visit in last 2 years, n (%)				
ED visit	266 805 (30.3)	158 118 (29.9)	108 687 (30.7)	**<.001**
Hospitalization	108 431 (12.3)	62 588 (11.8)	45 843 (13.0)	
None	506 529 (57.4)	307 574 (58.2)	198 955 (56.3)	

© 2024 Roumeliotis N et al. *JAMA Network Open*.

**Characteristic**	**Overall (N = 881 765)**	**Pre-COVID (n =528 280)**	**COVID-prevalent (n = 353 485)**	***P* value**
Triage level[g], n (%)				
Resuscitation	12 730 (1.4)	7 418 (1.4)	5 312 (1.5)	**<.001**
Emergent	299 875 (34.1)	175 252 (33.3)	124 623 (35.4)	
Urgent	407 170 (46.4)	244 217 (46.4)	162 953 (46.2)	
Less urgent (semi-urgent)	132 812 (15.1)	82 683 (15.7)	50 129 (14.2)	
Non-urgent	25 641 (2.9)	16 202 (3.1)	9 439 (2.7)	

Pre-COVID: April 1, 2016, to March 31, 2020; COVID-prevalent: April 1, 2020, to March 31, 2023.
ED: Emergency Department
*P-values are from chi-square test of independence between pre- and COVID-prevalent columns for both Hospitalizations and ED visits
a. Sex other n=798 (0.1%), not shown
b. Missing Urban/rural designation n= 59 840 (6.8%)
c. Missing Material deprivation for n=98 068 (11.1%)
d. ED data partially complete for Prince Edward Island (PEI), Nova Scotia (NS), Manitoba, Saskatchewan and British Columbia (BC); not mandated in Newfoundland (NF) and Labrador, New Brunswick (NB), the Northwest Territories and Nunavut.
e. Suicide and self-harm numbers for Quebec not included due to inconsistent coding
f. Defined as a tertiary center with a pediatric intensive care unit.
g. Triage level unknown for n=3537 (0.0%)

© 2024 Roumeliotis N et al. *JAMA Network Open*.

eTable 6. Characteristics of Mental Health Emergency Department Visits by Fiscal Year (N = 881,765)

	Fiscal Year						
**Characteristic**	**2016 - 2017**	**2017 - 2018**	**2018 - 2019**	**2019 - 2020**	**2020 - 2021**	**2021 - 2022**	**2022 - 2023**
Total	125 562	137 119	137 403	128 196	105 068	130 625	117 792
Sex[a], n (%)							
Female	74963 (59.7)	81756 (59.7)	83384 (60.7)	76850 (60.0)	66806 (63.6)	87514 (67.1)	77827 (66.2)
Male	50574 (40.3)	55260 (40.3)	53903 (39.3)	51237 (40.0)	38195 (36.4)	42958 (32.9)	39740 (33.8)
Age group, n (%)							
6-11 years	8292 (6.6)	9106 (6.6)	9837 (7.2)	9468 (7.4)	7535 (7.2)	9374 (7.2)	8064 (6.8)
12-17 years	56112 (44.7)	63654 (46.4)	63015 (45.9)	60491 (47.2)	53577 (51.0)	70442 (53.9)	62344 (52.9)
18-20 years	61158 (48.7)	64359 (46.9)	64551 (47.0)	58237 (45.4)	43956 (41.8)	50809 (38.9)	47384 (40.2)
Rural address[b], n (%)	19148 (16.7)	20710 (16.5)	21214 (16.9)	20195 (16.9)	17120 (16.9)	20619 (16.6)	19214 (17.3)
Material deprivation quintile[c], n (%)							
1 (less deprived)	20810 (18.8)	23492 (19.4)	22836 (18.9)	21713 (18.9)	19163 (19.7)	24862 (21.0)	18746 (18.8)
2	21386 (19.3)	23331 (19.2)	23707 (19.6)	22135 (19.2)	18906 (19.5)	23303 (19.6)	18275 (18.3)
3	21342 (19.3)	22960 (18.9)	23122 (19.1)	21746 (18.9)	18913 (19.5)	23055 (19.4)	19491 (19.5)
4	20968 (18.9)	23739 (19.6)	23189 (19.2)	22521 (19.6)	18320 (18.9)	22516 (19.0)	20034 (20.1)
5 (most deprived)	26176 (23.6)	27873 (23.0)	28155 (23.3)	27006 (23.5)	21830 (22.5)	24869 (21.0)	23207 (23.3)
Province/Territories[d], n (%)							
Alberta	19673 (15.7)	21321 (15.5)	21460 (15.6)	20125 (15.7)	18265 (17.4)	21965 (16.8)	19070 (16.2)
British Columbia	6109 (4.9)	6885 (5.0)	7360 (5.4)	7028 (5.5)	6394 (6.1)	7566 (5.8)	6566 (5.6)
Nova Scotia	1444 (1.2)	1566 (1.1)	1500 (1.1)	1360 (1.1)	1347 (1.3)	1704 (1.3)	1451 (1.2)
Ontario	55819 (44.5)	60666 (44.2)	59147 (43.0)	55832 (43.6)	45164 (43.0)	53609 (41.0)	48309 (41.0)
Prince Edward Island	265 (0.2)	255 (0.2)	291 (0.2)	277 (0.2)	292 (0.3)	319 (0.2)	297 (0.3)
Quebec	38561 (30.7)	41735 (30.4)	42739 (31.1)	38271 (29.9)	28921 (27.5)	40005 (30.6)	36687 (31.1)
Saskatchewan	3383 (2.7)	4409 (3.2)	4553 (3.3)	4949 (3.9)	4395 (4.2)	5118 (3.9)	5094 (4.3)
Yukon	308 (0.2)	282 (0.2)	353 (0.3)	354 (0.3)	290 (0.3)	339 (0.3)	318 (0.3)
Diagnostic subgroup, n (%)							
Anxiety	33065 (26.3)	34406 (25.1)	35184 (25.6)	35480 (27.7)	31801 (30.3)	36748 (28.1)	31089 (26.4)
Eating disorders	662 (0.5)	793 (0.6)	854 (0.6)	779 (0.6)	1471 (1.4)	2110 (1.6)	1528 (1.3)

© 2024 Roumeliotis N et al. *JAMA Network Open*.

Characteristic	2016 - 2017	2017 - 2018	2018 - 2019	2019 - 2020	2020 - 2021	2021 - 2022	2022 - 2023
Mood disorders	23034 (18.3)	26183 (19.1)	25814 (18.8)	24512 (19.1)	18513 (17.6)	24026 (18.4)	20663 (17.5)
Other mental disorders	20560 (16.4)	23510 (17.1)	24664 (18.0)	23220 (18.1)	19075 (18.2)	24746 (18.9)	22758 (19.3)
Personality disorders	5347 (4.3)	5761 (4.2)	5801 (4.2)	5255 (4.1)	4201 (4.0)	5285 (4.0)	5325 (4.5)
Schizophrenic/Psychotic	4841 (3.9)	4570 (3.3)	4440 (3.2)	4205 (3.3)	4156 (4.0)	4332 (3.3)	3929 (3.3)
Substance related disorders	23960 (19.1)	26100 (19.0)	25024 (18.2)	20531 (16.0)	12361 (11.8)	15538 (11.9)	16205 (13.8)
Suicide and self-harm[e]	14093 (11.2)	15796 (11.5)	15622 (11.4)	14214 (11.1)	13490 (12.8)	17840 (13.7)	16295 (13.8)
Transfer between centers, n (%)	24403 (19.4)	26584 (19.4)	26279 (19.1)	24422 (19.1)	23845 (22.7)	27554 (21.1)	24038 (20.4)
Admitted to a pediatric center[f], n (%)							
Yes	18884 (15.0)	21069 (15.4)	21856 (15.9)	22747 (17.7)	23449 (22.3)	32399 (24.8)	27278 (23.2)
No	106678 (85.0)	116050 (84.6)	115547 (84.1)	105449 (82.3)	81619 (77.7)	98226 (75.2)	90514 (76.8)
Mental health visit last 2 years, n (%)							
Emergency Department visit	36334 (28.9)	40655 (29.6)	41447 (30.2)	39682 (31.0)	33482 (31.9)	39177 (30.0)	36028 (30.6)
Hospitalization	15198 (12.1)	16102 (11.7)	16030 (11.7)	15258 (11.9)	13645 (13.0)	16512 (12.6)	15686 (13.3)
None	74030 (59.0)	80362 (58.6)	79926 (58.2)	73256 (57.1)	57941 (55.1)	74936 (57.4)	66078 (56.1)
Triage level[g], n (%)							
Resuscitation	1862 (1.5)	1755 (1.3)	1898 (1.4)	1903 (1.5)	1488 (1.4)	1902 (1.5)	1922 (1.6)
Emergent	40457 (32.3)	45827 (33.6)	45318 (33.2)	43650 (34.2)	36106 (34.5)	46416 (35.6)	42101 (35.9)
Urgent	58205 (46.5)	63919 (46.8)	63819 (46.7)	58274 (45.6)	48715 (46.5)	60374 (46.3)	53864 (45.9)
Less urgent (semi-urgent)	20262 (16.2)	20683 (15.2)	21543 (15.8)	20195 (15.8)	15669 (15.0)	18283 (14.0)	16177 (13.8)
Non-urgent	4290 (3.4)	4309 (3.2)	3955 (2.9)	3648 (2.9)	2810 (2.7)	3282 (2.5)	3347 (2.9)

a. Sex other/Unspecified n=798 (0.1%), not shown
b. Missing Urban/rural designation n= 59 840 (6.8%)
c. Missing Material Deprivation quintile for n=98 068 (11.1%)
d. ED data partially complete for Prince Edward Island (PEI), Nova Scotia (NS), Manitoba, Saskatchewan and British Columbia (BC); not mandated in Newfoundland (NF) and Labrador, New Brunswick (NB), the Northwest Territories and Nunavut.
e. Suicide and self-harm numbers for Quebec not included due to inconsistent coding
f. Defined as a tertiary center with a pediatric intensive care unit
g. Triage level unknown for n=3537 (0.0%)

© 2024 Roumeliotis N et al. *JAMA Network Open*.

eFigure 4. Rates of Mental Health Emergency Department Visits by 10 000 Person-Years for Each Diagnostic Subgroup Stratified by Provinces (With Complete ED Reporting)

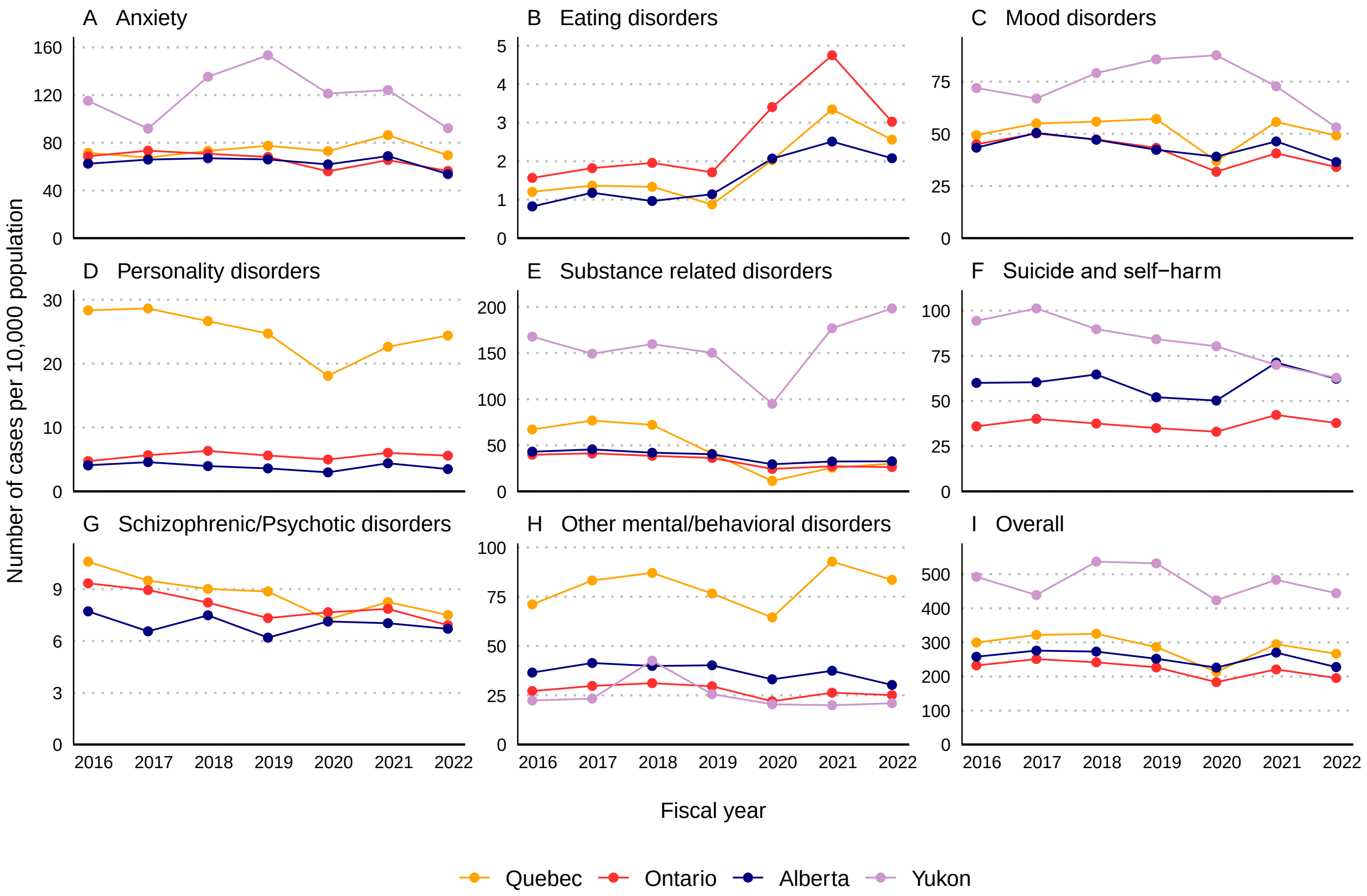

ED: Emergency Department
Points correspond to aggregate data by fiscal year, and are displayed at the beginning of the fiscal year.
Low counts for Yukon territory

© 2024 Roumeliotis N et al. *JAMA Network Open*.

eTable 7. Emergency Department Visit Rate by 10 000 Person-Years, for Mental Health Disorders Pre–COVID-19 Period vs COVID-19–Prevalent Period (Ontario, Quebec, Alberta, and Yukon) by Sex

Diagnostic subgroup	Pre-COVID	COVID-prevalent	Rate ratio (95% CI)
**Anxiety**	70.07	64.63	0.92 (0.91, 0.93)
Male	50.40	41.00	0.81 (0.80, 0.83)
Female	90.67	89.32	0.99 (0.97, 1.00)
**Eating disorders**	1.47	3.13	2.13 (2.03, 2.23)
Male	0.33	0.48	1.45 (1.24, 1.70)
Female	2.67	5.91	2.21 (2.10, 2.33)
**Mood**	48.61	39.86	0.82 (0.81, 0.83)
Male	31.66	22.62	0.71 (0.70, 0.73)
Female	66.37	57.88	0.87 (0.86, 0.88)
**Personality**	11.51	9.93	0.86 (0.84, 0.88)
Male	6.85	5.28	0.77 (0.74, 0.80)
Female	16.40	14.78	0.90 (0.88, 0.92)
**Schizophrenic/Psychotic**	8.50	7.45	0.88 (0.85, 0.90)
Male	11.38	9.45	0.83 (0.81, 0.86)
Female	5.49	5.35	0.98 (0.93, 1.02)
**Substance related**	47.02	26.13	0.56 (0.55, 0.56)
Male	45.31	24.66	0.54 (0.53, 0.55)
Female	48.80	27.67	0.57 (0.56, 0.58)
**Suicide and self-harm**[a]	42.56	43.56	1.02 (1.01, 1.04)
Male	29.00	25.48	0.88 (0.86, 0.90)
Female	56.79	62.48	1.10 (1.08, 1.12)
**Other**[b]	45.61	42.41	0.93 (0.92, 0.94)
Male	38.59	28.05	0.73 (0.71, 0.74)
Female	52.97	57.42	1.08 (1.07, 1.10)

Gray shaded lines highlight a significant increase in Emergency Department (ED) visit rate during COVID-prevalent period
Provinces reported above have complete ED reporting
CI: Confidence interval
a. For suicide and self-harm only Alberta, Ontario et Yukon included.
b. Other includes adjustment disorder, dissociative disorders, Attention deficit and hyperactivity, tic disorders and other behavioural disorders

© 2024 Roumeliotis N et al. *JAMA Network Open*.
