# Supplementary material for: Mental Health Hospitalizations in Canadian Children, Adolescents, and Young Adults Over the COVID-19 Pandemic
Source: JAMA Netw Open. 2024 Jul 8;7(7):e2422833. doi: 10.1001/jamanetworkopen.2024.22833 (PMC11231797; doi:10.1001/jamanetworkopen.2024.22833)
Supplement: Supplement 3. — Data Sharing Statement [file jamanetwopen-e2422833-s003.pdf]

Data Sharing Statement

Roumeliotis. Mental Health Hospitalizations in Canadian Children, Adolescents, and Young Adults Over the COVID-19 Pandemic. *JAMA Netw Open*. Published July 08, 2024. doi:10.1001/jamanetworkopen.2024.22833

Data
Data available: No

Additional Information
Explanation for why data not available: Data from the Study is provided by the health administrative database of Canadian Institute for Health Information (CIHI). CIHI will not release record level data to respect the Canadian privacy act. Aggregate data can be provided on request, and with agreement from CIHI.
